# Asymmetric Deactivation of HIV-1 gp41 following Fusion Inhibitor Binding

**DOI:** 10.1371/journal.ppat.1000674

**Published:** 2009-11-26

**Authors:** Kristen M. Kahle, H. Kirby Steger, Michael J. Root

**Affiliations:** Department of Biochemistry and Molecular Biology, Kimmel Cancer Center, Thomas Jefferson University, Philadelphia, Pennsylvania, United States of America; Harvard Medical School, United States of America

## Abstract

Both equilibrium and nonequilibrium factors influence the efficacy of pharmaceutical agents that target intermediate states of biochemical reactions. We explored the intermediate state inhibition of gp41, part of the HIV-1 envelope glycoprotein complex (Env) that promotes viral entry through membrane fusion. This process involves a series of gp41 conformational changes coordinated by Env interactions with cellular CD4 and a chemokine receptor. In a kinetic window between CD4 binding and membrane fusion, the N- and C-terminal regions of the gp41 ectodomain become transiently susceptible to inhibitors that disrupt Env structural transitions. In this study, we sought to identify kinetic parameters that influence the antiviral potency of two such gp41 inhibitors, C37 and 5-Helix. Employing a series of C37 and 5-Helix variants, we investigated the physical properties of gp41 inhibition, including the ability of inhibitor-bound gp41 to recover its fusion activity once inhibitor was removed from solution. Our results indicated that antiviral activity critically depended upon irreversible deactivation of inhibitor-bound gp41. For C37, which targets the N-terminal region of the gp41 ectodomain, deactivation was a slow process that depended on chemokine receptor binding to Env. For 5-Helix, which targets the C-terminal region of the gp41 ectodomain, deactivation occurred rapidly following inhibitor binding and was independent of chemokine receptor levels. Due to this kinetic disparity, C37 inhibition was largely reversible, while 5-Helix inhibition was functionally irreversible. The fundamental difference in deactivation mechanism points to an unappreciated asymmetry in gp41 following inhibitor binding and impacts the development of improved fusion inhibitors and HIV-1 vaccines. The results also demonstrate how the activities of intermediate state inhibitors critically depend upon the final disposition of inhibitor-bound states.

## Author Summary

Blocking HIV-1 entry into host cells is a viable strategy to prevent viral infection. Entry is mediated by viral surface proteins gp120 and gp41, which undergo large structural changes upon encountering the host cell. One critical structural change involves the association of two distinct gp41 regions, denoted N-HR and C-HR. Previous work demonstrated that antivirals targeting the N-HR and C-HR physically obstruct the association of these gp41 regions, thereby preventing HIV-1 entry. Here, we show that these inhibitors work through an additional mechanism—they induce the irreversible deactivation of gp41. Furthermore, the antiviral potency of these inhibitors is significantly influenced by the speed of this irreversible deactivation. When an inhibitor binds the C-HR, gp41 rapidly deactivates before the inhibitor can efficiently dissociate. By contrast, when an inhibitor binds the N-HR, gp41 deactivates very slowly, allowing sufficient time for inhibitor dissociation and resumption of gp41 structural changes. The disparity in the rate of deactivation induced by N-HR- and C-HR-targeting inhibitors reflects a fundamental difference in deactivation mechanism. Thus, the results point to an unappreciated asymmetry in gp41 following inhibitor binding. Our findings are relevant to the development of future entry inhibitors of HIV-1 and potentially other viruses that utilize similar entry mechanisms.

## Introduction

**Figure 1:**
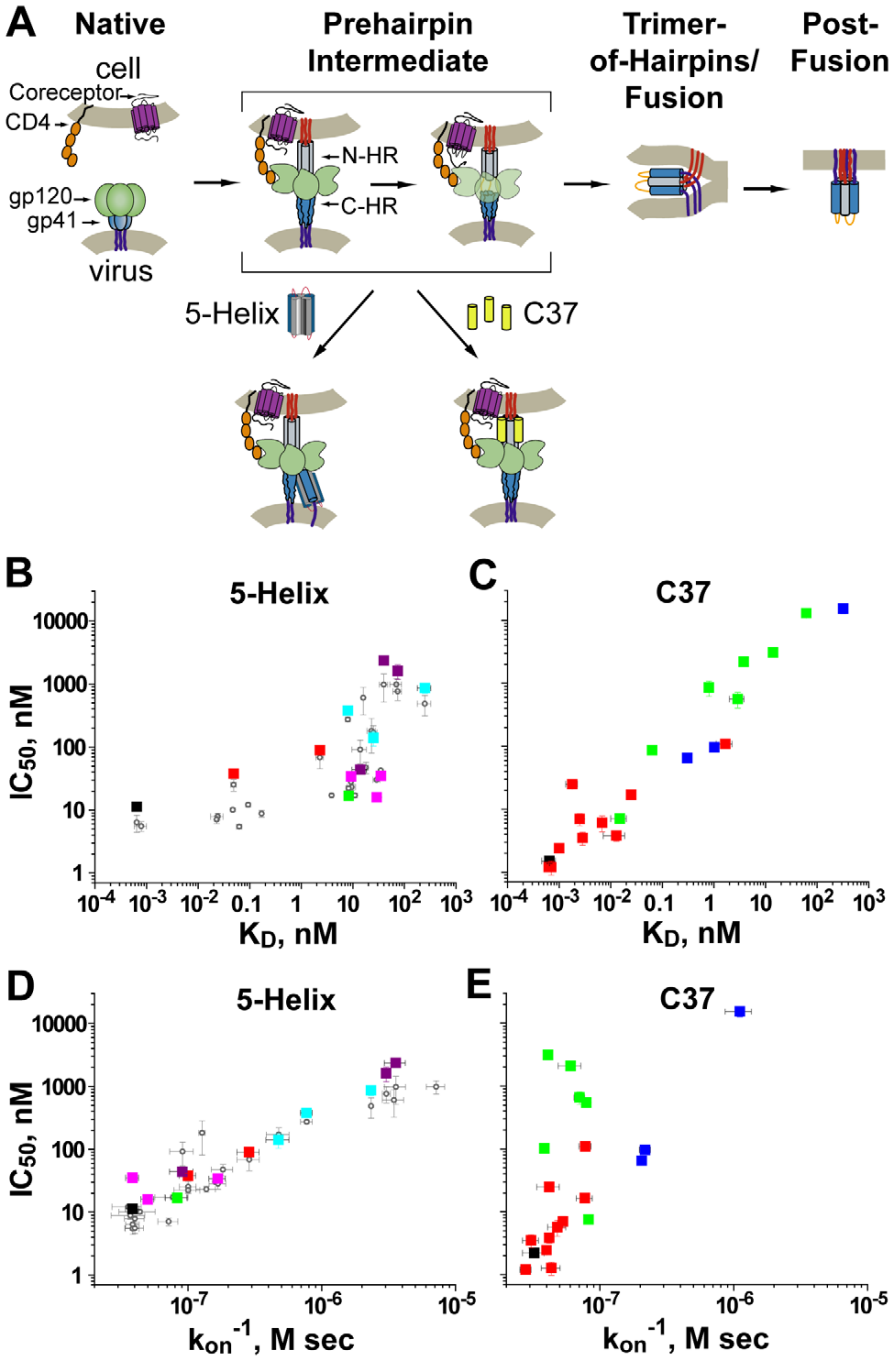
Inhibition of HIV-1 membrane fusion. (A) A working model of HIV-1 entry. Env subunit gp120 (green) interacts with cellular CD4 (orange), triggering gp41 to extend its N-terminus (red) toward the target cell membrane. Subsequent binding of gp120 to a chemokine receptor (labeled coreceptor, purple) leads to collapse of the ectodomain into a trimer-of-hairpins and juxtaposition of viral and cellular membranes required for fusion. Fusion inhibitors C37 and 5-Helix respectively bind the gp41 N-HR (gray) and C-HR (blue) segments transiently exposed during the extended prehairpin state. (B–E) Affinity and kinetic dependence to 5-Helix (B, D) and C37 (C, E) inhibition. For series of inhibitor variants with mutations in their gp41 binding sites, IC50 values are plotted as a function of K_D_ or the inverse of k_on_. Each square represents a different inhibitor variant and are color coded according to mutation class (see Tables S1 and S2). Gray circles in panels B and D correspond to inhibitory activities of 5-Helix variants from previously reported cell-cell fusion experiments [3]. Please note that the axes of these plots are in logarithmic scale.

Intermediate states of biological processes are increasingly common targets for inhibition [1],[2]. The transient nature of such targets makes inhibitory potency a complex function of both equilibrium and nonequilibrium factors [3]. Here, we characterize the intermediate-state inhibition of HIV-1 gp41, part of the Env glycoprotein complex that mediates viral entry through membrane fusion. The process is coordinated by sequential binding of Env subunit gp120 to cellular CD4 and a chemokine receptor such as CXCR4 or CCR5 (Figure 1A) [4]. These events trigger rearrangements of the gp41 ectodomain that culminate in formation of a compact structure known as the trimer-of-hairpins (TOH) [5],[6]. Molecules that block TOH formation can effectively inhibit HIV-1 membrane fusion both *in vitro* and *in vivo*.

The primary targets for gp41 inhibitors are two heptad repeat (HR) segments in the N- and C-terminal regions of the gp41 ectodomain (denoted N-HR and C-HR, respectively) [7]. In the fusogenic TOH conformation, these HR regions form a stable bundle of six α-helices: N-HR segments from three gp41 ectodomains form a trimeric coiled coil, around which the three C-HR segments pack in an antiparallel manner into hydrophobic grooves on the coiled-coil surface [5],[6]. Inhibitors bind the N-HR or C-HR segment prior to bundle formation and prevent collapse of gp41 into its TOH conformation [4]. The best characterized are linear peptides derived from the C-HR and adjacent regions of the gp41 ectodomain [7]–[9]. Denoted C-peptides, these agents target the N-HR in its coiled-coil conformation, binding the same hydrophobic grooves that would normally interact with gp41 C-HR segments [10],[11]. One C-peptide, T20 (enfuvirtide) effectively suppresses HIV-1 infection in humans and is currently used as salvage therapy for AIDS patients refractory to other antiviral medications [12],[13]. In a complementary manner, engineered proteins that structurally mimic all or part of the N-HR coiled coil can inhibit HIV-1 entry by binding the gp41 C-HR segments [14],[15]. A well characterized example is the 5-Helix protein, which contains all three N-HR segments but only two C-HR segments; when properly folded, 5-Helix exposes a single C-peptide binding site that strongly interacts with gp41 C-HR regions [16].

C-peptides and 5-Helix do not interact with the native state of Env prior to gp120/CD4 interaction [3],[17],[18]. Rather, these inhibitors target an intermediate state that exists in a kinetic window between gp120/CD4 binding and TOH formation [19]–[21]. Evidence suggests that the gp41 ectodomain in this transient prehairpin state adopts an extended conformation, with its N-terminus (called the fusion peptide) inserted in the target cell membrane, its transmembrane region embedded in the viral membrane, and its N-HR coiled coil and C-HR segments exposed to bulk solution (Figure 1A) [22]. Despite the kinetic restrictions of targeting a transient conformation, gp41 inhibitors can possess potent (low nanomolar to high picomolar) antiviral activity [8],[23].

Because gp41 inhibitors target a transient intermediate state, their potency is not simply determined by equilibrium binding affinity [3],[19],[21]. Nonequilibrium parameters, such as the rate of inhibitor association and the lifetime of the intermediate state also influence the degree of inhibition. Previously, we showed that the potency of 5-Helix was primarily determined by these kinetic properties: for a series of 5-Helix variants with mutations in their gp41 binding sites, IC50 values varied inversely with association rate constants (k_on_), but showed poor correlation with equilibrium dissociation constants (K_D_) [3]. Here, we unexpectedly found that the opposite relationship held true for a C-peptide inhibitor: IC50 values for a series of C37 variants depended in large part on binding affinity, but did not correlate with k_on_. Thus, despite targeting the same intermediate state, the physical properties underlying 5-Helix and C37 inhibition were fundamentally different. We linked this discrepancy to the ultimate disposition of Env following inhibitor binding and employed this knowledge to design novel C37 inhibitors that retained potent antiviral activity in the setting of C-peptide escape mutations.

## Results

### Physical Properties of 5-Helix and C37 Inhibition

We investigated how the antiviral potencies of 5-Helix and C37 inhibition were impacted by Ala and Asp substitutions at residue positions that contact gp41. Concurrently, we used a bimolecular 5-Helix/C37 interaction assay to measure the effect of mutations on the binding affinities and association rates of these inhibitor variants (see Materials and Methods, Figure S1). In a previous study employing cell-cell fusion experiments, IC50 values for a series of 5-Helix variants poorly correlated with K_D_ but showed a strong inverse dependence on k_on_ [3]. Here, we found the same behavior was quantitatively maintained in viral infectivity assays (Figure 1B, D; Table S1). Specifically, the degree of affinity disruption caused by the mutations was not predictive of 5-Helix antiviral activity. For instance, 5-Helix and 5-Helix_V549A/L556A/Q563A_ had very similar IC50 values (11 and 16 nM, respectively) despite the 50,000-fold difference in their K_D_ values (0.00065 and 29 nM, respectively). Furthermore, IC50 values for 5-Helix_V549A/L556A/Q563A_ (16 nM) and 5-Helix_V549D/L556A_ (2400 nM) were very different, even though their K_D_ values were comparable (29 versus 40 nM, respectively). Rather, we found that antiviral activity closely tracked with inhibitor association rate: potent inhibitors 5-Helix and 5-Helix_V549A/L556A/Q563A_ shared similarly high k_on_ values (∼3×10^7^ M^−1^ sec^−1^), while the weak inhibitor 5-Helix_V549D/L556A_ exhibited a 100-fold lower k_on_ value (0.028×10^7^ M^−1^ sec^−1^). Thus, more rapidly associating 5-Helix variants had lower IC50 values, independent of binding affinity. The data implied that 5-Helix inhibition is kinetically restricted by the short exposure of its C-HR binding site: more rapidly associating variants are more likely to bind gp41 during the kinetic window of C-HR exposure. This information enabled us to estimate that the C-HR is exposed for a few seconds during the prehairpin intermediate state [3].

Because C37 inhibits the same intermediate state through a complementary mechanism, we expected that its antiviral potency should be similarly correlated with k_on_, and not K_D_. For a series of C37 variants, IC50 values determined in viral infectivity assays spanned more than four orders-of-magnitude, from 1.2 nM to 15 µM (Table S2, Figure S1). A similarly large range in K_D_ values (0.65 pM to 320 nM) was also measured. Contrary to our expectations, generally good correlation was observed between IC50 and K_D_ values over this entire range (Figure 1C). By contrast, C-peptide mutations had comparatively little impact (<30-fold) on the rate of C37 association. Consequently, poor correlation was observed between IC50 and k_on_ values (Figure 1E). Thus, C37 potency is primarily determined by equilibrium binding affinity, in stark contrast to the kinetic dependence of 5-Helix inhibition.

**Figure 2:**
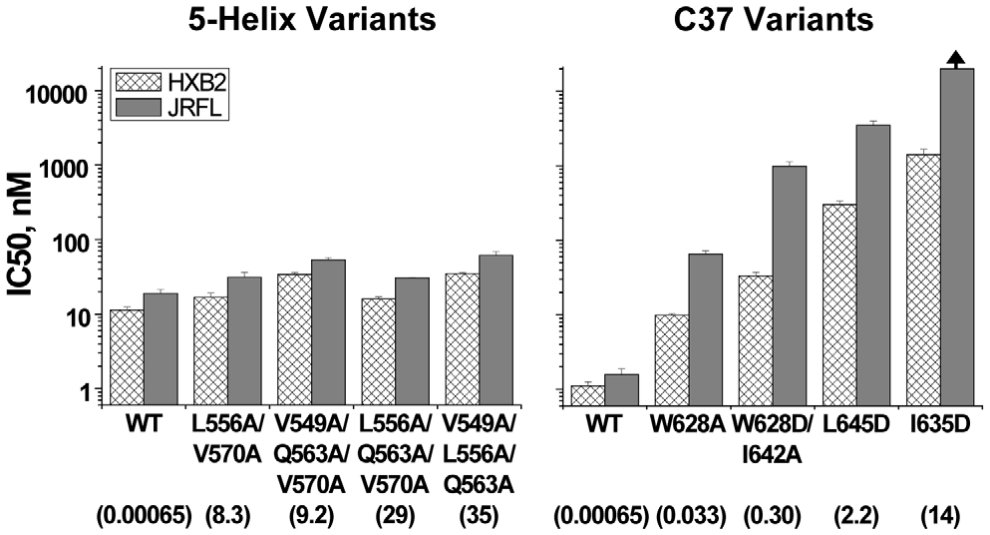
5-Helix and C37 inhibition of primary isolate HIV-1 strain JR-FL. IC50 values for the wild type and lower affinity variants were determined for HIV-1_JR-FL_ infections of HOS-CD4-CCR5 cells (gray bars). For comparison, the IC50 values for HIV-1_HXB2_ infections of HOS-CDR-CXCR4 cells are also shown (hatched bars). The numbers below the axis labels are K_D_ values (in nM) measured for binding to the HXB2 sequence. The arrow indicates an IC50 value in excess of 20 µM.

The disparity in the physical properties of C37 and 5-Helix inhibition was not dependent on HIV-1 Env strain or coreceptor utilization. The data in Figure 1 were generated using the CXCR4-tropic, laboratory-adapted Env_HXB2_, but qualitatively similar results were obtained with the CCR5-tropic, primary isolate Env_JR-FL_: the potency of rapidly-associating 5-Helix inhibitors was largely unaffected by affinity disruption, while the potency of C37 variants was significantly reduced (Figure 2). A dependence of inhibitory potency on binding strength has also been observed for small D-peptides [24] and a monoclonal antibody [25] that bind a deep hydrophobic pocket on the N-HR coiled coil. Thus, affinity-dependent inhibitory potency appears to be a general property of N-HR targeting inhibitors, irrespective of inhibitor size and chemical nature. This contrast with the kinetic properties of 5-Helix inhibition points to a fundamental mechanistic difference between N-HR- and C-HR-targeting gp41 inhibitors.

### Model of Intermediate-State Inhibition

**Figure 3:**
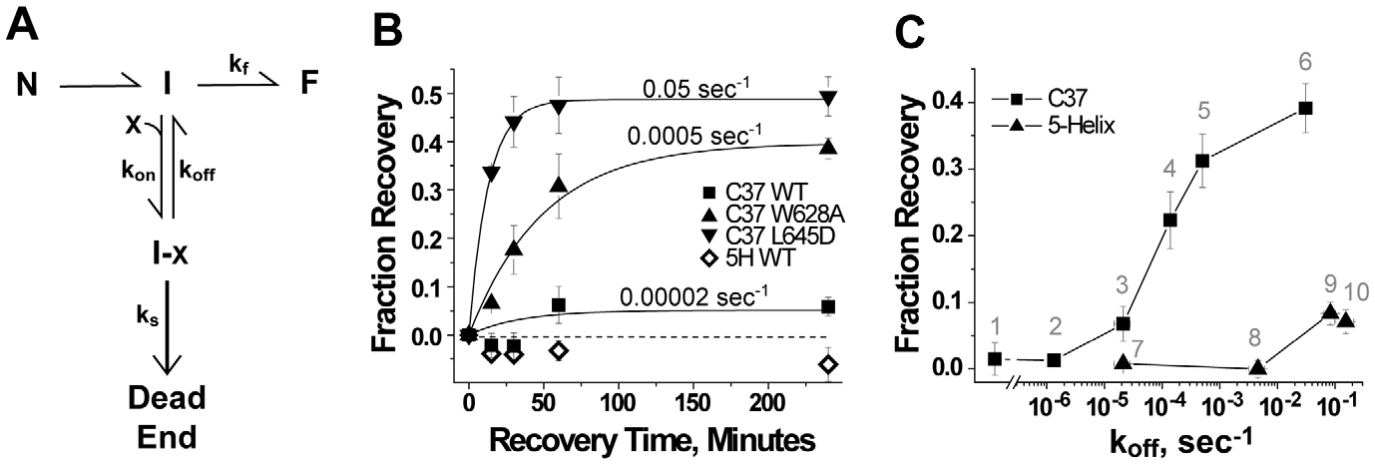
Reversibility of gp41 inhibition. (A) Schematic of intermediate-state inhibition by C37 and 5-Helix. States and rate constants are defined in the text. (B) Time course of recovery from C37 and 5-Helix inhibition. HIV-1 was preincubated 2 hours with target cells in the presence of high inhibitor concentrations to trap gp41 in the inhibitor-bound state (I-X in Panel A). At time t = 0, cells were rapidly washed with virus-free, inhibitor-free media to allow inhibitor dissociation and recovery of fusion activity. Infection was terminated by the addition of 1 µM C37 at various times following this wash. The number above each C37 data set is the k_off_ value for that C-peptide variant. (C) Dependence of reversibility on inhibitor k_off_. Fraction recovery after 1 hour is plotted as a function of k_off_ for C37 (square) and 5-Helix (triangle) variants numbered as follows: (1) di-C37, (2) C37-KYI, (3) C37 WT, (4) C37_I635A_, (5) C37_W628A_, (6) C37_L645D_, (7) 5-Helix WT, (8) 5-Helix_L556A/Q563A_, (9) 5-Helix_L556A/V570A_, (10) 5-Helix_L556A/Q563D_. The data represent the mean ± SEM of three independent experiments.

Affinity-dependent inhibition by C37 implies that the C-peptide/gp41 interaction is reversible, mimicking an equilibrium process. By contrast, the kinetic dependence to 5-Helix inhibition implies that the 5-Helix/gp41 interaction is functionally irreversible, as if 5-Helix association triggers rapid deactivation of gp41 before the inhibitor can dissociate. To account for the different inhibitory properties of C37 and 5-Helix, we developed a single quantitative model of intermediate state inhibition shown in Figure 3A (hereafter denoted Scheme 1). Here, N, I and F symbolize the native, intermediate, and the fusogenic conformations of Env, respectively. The rate constants k_on_ and k_off_ describe the kinetics of inhibitor (X) binding to I, where the equilibrium dissociation constant K_D_ equals k_off_/k_on_. The constant k_f_ refers to the unidirectional rate out of I and governs the lifetime of this intermediate state. The constant k_s_ describes the rate of irreversible deactivation of the inhibitor-bound gp41 (I-X). Based on this simple model of intermediate state inhibition, an equation describing the IC50 value can be derived [3]:
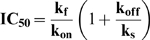
(Equation 1)


In the case when inhibitor dissociation is much slower than gp41 deactivation (k_off_<<k_s_), the equation predicts that the IC50 values will vary inversely with the inhibitor association rate (IC50 ≅ k_f_/k_on_). The dependence solely on k_on_ is logical because virtually every inhibitor association event leads to irreversible deactivation. At the other extreme, when inhibitor dissociation occurs much more rapidly than gp41 deactivation (k_off_>>k_s_), the second term in Equation 1 predominates, and the IC50 values depend on binding strength (IC50 ≅ k_f_ K_D_/k_s_, where the ratio k_off_/k_on_ was replaced with K_D_). The dependence on K_D_ in this situation is due to the ability of the inhibitor to associate and dissociate numerous times (the definition of equilibrium binding) before gp41 commits irreversibly toward either its fusogenic or dead-end conformation.

Quantitative fits of IC50, K_D_ and k_on_ data to Equation 1 were most remarkable for a 200-fold disparity in the deactivation rate (k_s_) for C37 and 5-Helix inhibition (k_s-C37_ = 0.00049 sec^−1^; k_s-5H_ = 0.11 sec^−1^; Figure S2). The data suggest that the C37-bound intermediate state can persist in an inhibited but recoverable form for an average of ∼35 minutes, longer than the average bound lifetime for most C37 variants (Table S2). By contrast, 5-Helix-bound gp41 appears to undergo irreversible deactivation in ∼10 seconds, shorter than the dissociation time constant for nearly all 5-Helix variants (Table S1). Thus, the different inhibitory properties of C37 and 5-Helix appear to reflect two extremes in the fate of Env following inhibitor binding.

### Recovery from Intermediate-State Inhibition

Our model predicts that C37 inhibition of HIV-1 entry is reversible if the peptide dissociates before gp41 deactivates. To test this prediction, we developed an inhibitor-washout viral infectivity assay. HIV-1 infection was carried out in the presence of high inhibitor concentration (>IC95) to trap CD4-engaged Env in the inhibitor-bound intermediate state (I-X in Scheme 1). Following this pre-incubation, culture media was rapidly exchanged with inhibitor-free, virus-free solution that contained an anti-CD4 antibody to block new activation of any unengaged Env. Successful viral infection, therefore, required inhibitor dissociation from gp41 trimers that had not undergone deactivation. Fusion activity recovered only slightly from inhibition by wild type C37, which has a small k_off_ compared to the gp41 deactivation rate k_s-C37_ (0.00049 sec^−1^) (Figure 3B). However, fusion activity showed greater and more rapid rebound for C37 variants with larger k_off_ values. This behavior reflects a kinetic competition between C37 dissociation and gp41 deactivation: as k_off_ increases, the likelihood (and rate) of inhibitor dissociation increases, resulting in greater (and faster) recovery of fusion activity.

Similar recovery of fusion activity was observed in inhibitor-washout experiments employing PIE7, a small D-peptide that targets the gp41 N-HR coiled coil (Figure S4). These data support our conclusions that gp41 bound to an N-HR inhibitor persists for an extended duration prior to deactivation. The reversibility of C-peptide and D-peptide inhibition starkly contrasts the lack of recovery from 5-Helix blockade (Figure 3B, C). The apparent k_s-5H_ (0.11 sec^−1^) is much greater than the k_off_ values for the two 5-Helix proteins that showed no fusion recovery in the washout assay. For these molecules, inhibitor-bound Env is much more likely to deactivate before 5-Helix dissociates. Even for 5-Helix variants with k_off_ values close to 0.1 sec^−1^, only a small amount of recovery was observed, perhaps because most inhibitor-bound Env had already deactivated before these 5-Helix variants were washed away.

### Design of “Irreversible” C37 Variants

**Figure 4:**
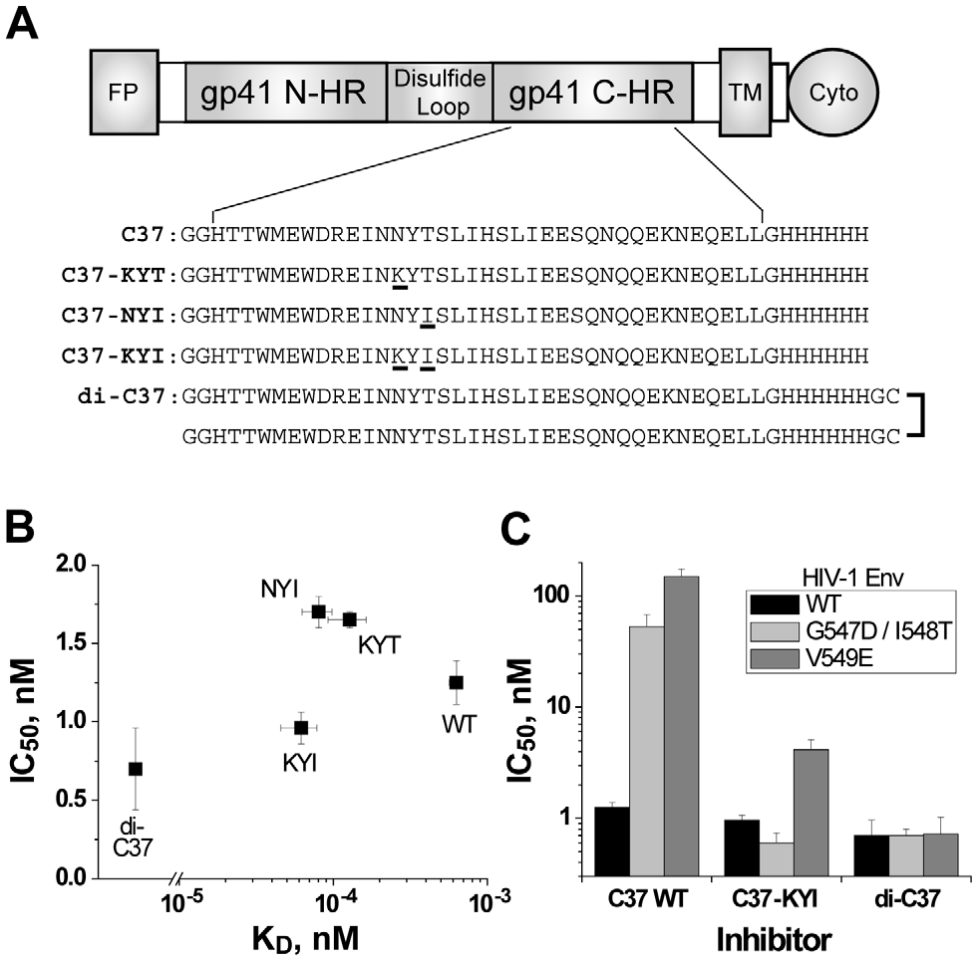
Inhibitory activity of tighter-binding C37 variants. (A) Schematic of gp41 depicting the N-HR and C-HR segments, fusion peptide (FP), transmembrane (TM), and cytoplasmic (Cyto) domains. The wild type C37 sequence below the diagram is derived from C-HR residues 625–661 of HIV-1_HXB2_ gp41. The sequences of kinetically restricted variants C37_N637K_ (KYT), C37_T639I_ (NYI), C37_N637K/T639I_ (KYI) are also shown with mutated residues underlined. In the dimeric construct di-C37, two wild type peptides are crosslinked through C-terminal Cys residues. (B) Potency of high affinity C37 variants against wild type HIV-1. IC50 data are plotted as a function of K_D_ for wild type C37 (WT), C37-KYT, C37-NYI, C37-KYI and di-C37. (C) Effect of affinity-disrupting N-HR mutations on C37 potency. IC50 values for C37 WT, C37-KYI, and di-C37 were determined for wild type Env (black), Env_G547D/I548T_ (light gray) and Env_V549E_ (dark gray).

Inhibition by high affinity 5-Helix variants is functionally irreversible because gp41 deactivation occurs much more rapidly than inhibitor dissociation. To test if extremely high affinity C-peptides are similarly irreversible, we engineered four C37 variants that bound more tightly to the gp41 N-HR coiled coil than wild type C37 (Figure 4A). Three have point mutations in the Asn637/Thr639 locus previously shown to enhance binding affinity 5- to 10-fold (HKS and MJR, submitted). The fourth is a dimeric C37 variant (denoted di-C37) that makes multivalent interactions with the N-HR coiled coil. We observed no recovery from inhibition by these tighter binding C-peptides, consistent with their reduced k_off_ values (Figure 3C). According to our model, such high affinity peptides should be kinetically restricted inhibitors like 5-Helix; that is, their potencies should be largely independent of binding affinity (Equation 1 with k_off_<<k_s_). Indeed, against wild type HIV-1, IC50 values for these engineered peptides (0.7 to 1.7 nM) were not significantly different from the wild type C37 IC50 (Figure 4B). Moreover, the antiviral activities of C37_N637K/T639I_ (C37-KYI) and di-C37 were much less sensitive to gp41 N-HR mutations that disrupt inhibitor binding affinity. While the G547D/I548T and V549E substitutions [26]–[28] conferred 40- to 120-fold resistance to wild type C37, they had minimal impact on the potency of C37-KYI and di-C37 (Figure 4C). Hence, binding strength plays a greatly diminished role in determining the potency of kinetically restricted C37 variants.

### Impact of Chemokine-Receptor Binding on the Deactivation of Inhibitor-Bound gp41

**Figure 5:**
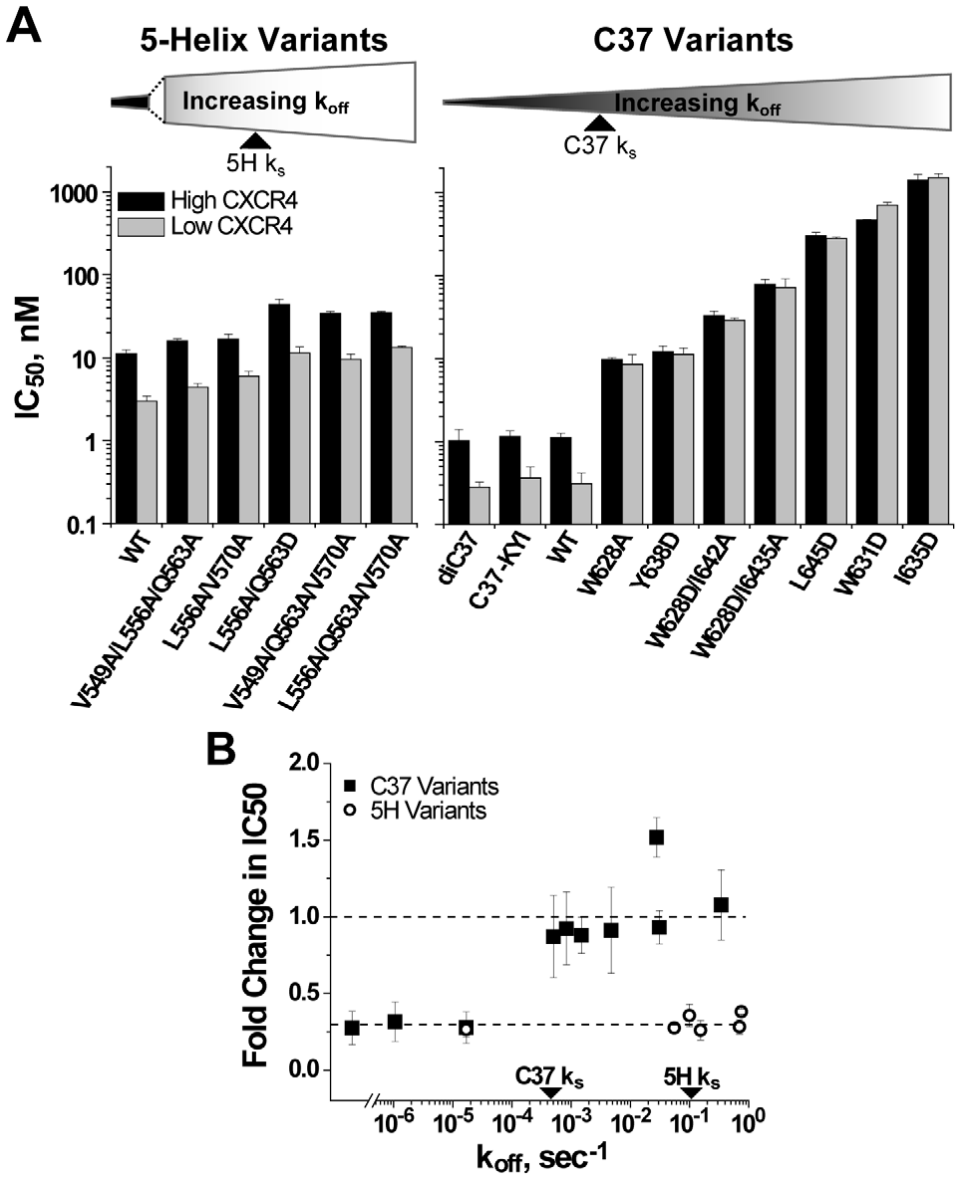
Effect of chemokine receptor density on 5-Helix- and C37-inhibitory activity against HIV-1_HXB2_. (A) Comparison of IC50 values determined utilizing target cells expressing high (black) or low (gray) levels of CXCR4. Inhibitors are ordered according to increasing k_off_ values. (B) Ratio of the low-CXCR4 IC50 to the high-CXCR4 IC50 plotted as a function of inhibitor k_off_. Each data point reflects a unique C37 (squares) or 5-Helix (circles) variant with error formally propagated. The gp41 deactivation rates (k_s_) for C37 and 5-Helix inhibition are indicated for comparative purposes.

Chemokine-receptor levels on target cells influence the kinetic properties of HIV-1 membrane fusion and the potency of gp41 inhibition [3],[19],[21]. We speculated that these levels might also influence the rate of gp41 deactivation following inhibitor binding. We compared C37 and 5-Helix inhibitory activity against HIV-1_HXB2_ using target cells expressing low and high amounts of CXCR4 (Figure 5). Lowering CXCR4 levels led to a 3.5-fold increase in potency for the kinetically restricted inhibitors di-C37, C37-KYI, and wild type 5-Helix. For 5-Helix inhibition, this enhancement in potency was maintained for all variants, including ones with k_off_ values in excess of k_s-5H_. By contrast, IC50 values were independent of CXCR4 levels for C37 variants with k_off_ values equal to or in excess of k_s-C37_. Qualitatively similar results were obtained with HIV-1_Ba-L_ using target cells expressing different levels of CCR5 (Figure S5), suggesting that the observed behaviors are general properties of C37 and 5-Helix inhibition.

Reducing surface expression of chemokine receptors slows down Env-mediated membrane fusion, prolonging the average lifetime of the inhibitor-sensitive intermediate [19]. The effect increases the opportunity for C37 and 5-Helix to bind, thereby potentiating inhibition. For kinetically restricted inhibitors, this potentiation leads to enhanced potency (IC50 ≅ k_f_/k_on_). For rapidly dissociating inhibitors, however, this potentiation is modified by any changes to the deactivation of inhibitor-bound gp41 (IC50 ≅ k_f_ K_D_/k_s_). For these inhibitors, the dependence of potency on both k_f_ and k_s_ reflects the likelihood that the bound-state deactivates before the unbound state progresses toward fusion. For 5-Helix inhibition, the degree of potentiation observed for the wild type inhibitor is maintained for low affinity variants, suggesting that reducing chemokine-receptor levels does not alter k_s-5H_. Conversely, inhibitor potentiation is lost for low affinity C37 variants, implying that lowering chemokine receptor levels slows both k_f_ and k_s-C37_ to the same degree. Thus, these data strongly suggest that deactivation of C37-bound gp41 involves chemokine-receptor binding, while deactivation of 5-Helix-bound gp41 proceeds through a completely different, chemokine receptor-independent mechanism.

### Asymmetric Exposure of the gp41 N-HR and C-HR

**Figure 6:**
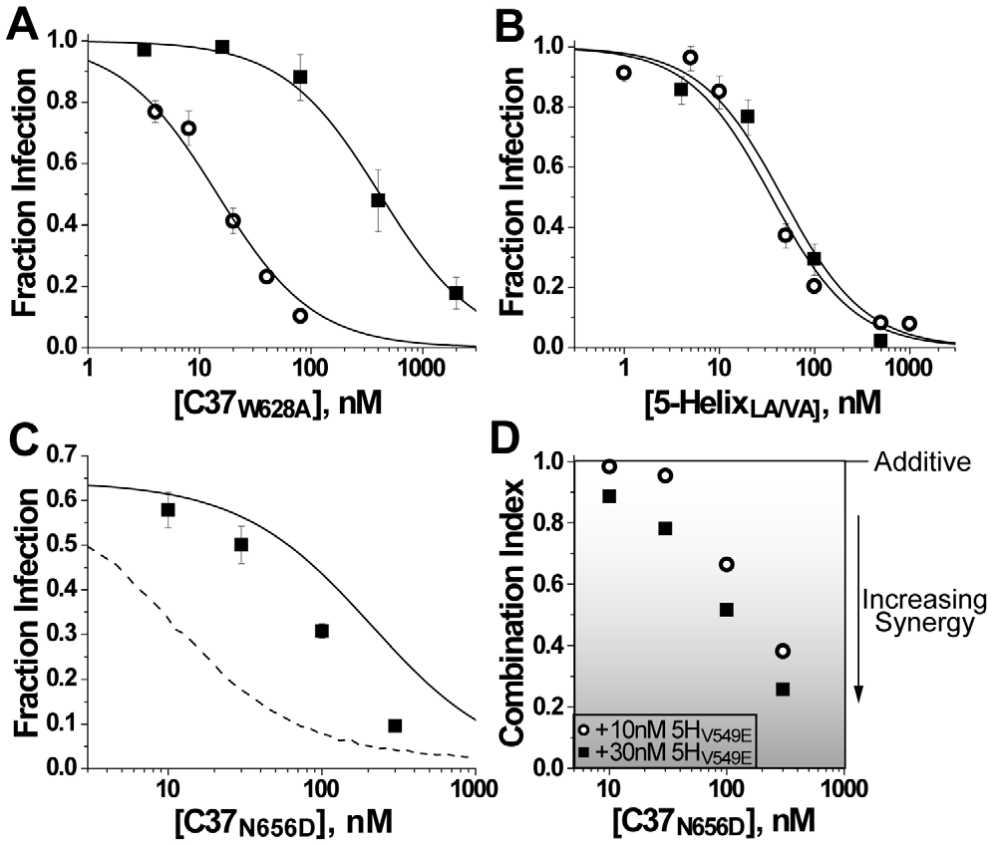
Overlap of the C37- and 5-Helix-sensitive intermediate states. (A) C37_W628A_ inhibitory activity against HIV-1 by standard assay (squares) or in a 5-Helix-washout assay after Env was first trapped in the 5-Helix_L556A/V570A_-bound state (circles). In the standard assay, C37_W628A_ was coincubated with virus and cells at the beginning of infection. In the 5-Helix-washout assay, target cells preincubated with HIV-1 and 5-Helix_L556A/V570A_ were washed with media containing C37_W628A_. (B) 5-Helix_L556A/V570A_ inhibitory activity against HIV-1 by standard assay (squares) or in a C37-washout assay after Env was first trapped in the C37_W628A_-bound state (circles). (C) Antiviral activity of C37_N656D_ in the presence of 30 nM 5-Helix_V549E_. The individual IC50 values for C37_N656D_ and 5-Helix_V549E_ were determined to be 130±10 nM and 54±2 nM, respectively. The solid line denotes the titration expected if C37 and 5-Helix target separate intermediate states (additive inhibition). The dotted line denotes the titration expected if C37 and 5-Helix could bind simultaneously to the same intermediate state (synergistic inhibition). These different binding scenarios are depicted schematically in Figure S7, and their quantitative evaluations are presented in Text S1. (D) Concentration dependence to synergistic inhibitory activity. Combination indices were calculated following the method of Chou and Talalay [49] for inhibition experiments performed with C37_N656D_ and either 10 nM (circles) or 30 nM (squares) 5-Helix_V549E_. A diminishing combination index below unity indicates increasing synergistic activity.

The differences in deactivation of C37- and 5-Helix-bound gp41 led us to question whether these inhibitors actually target the same intermediate state during viral entry. To test if the N-HR and C-HR segments were exposed simultaneously, we explored how well C37 inhibited when fusion was first trapped in the 5-Helix-bound intermediate state. Using 5-Helix_L556A/V570A_ (a 5-Helix variant that exhibited appreciable recovery from inhibition—label 9 of Figure 3C), we performed a 5-Helix-washout viral infectivity assay as previously described, except that C37 or C37_W628A_ was included in the washout solution. C-peptide potency was improved 10- to 30-fold in these inhibitor-washout experiments compared to standard inhibition experiments (Figure 6A, Figure S6A). Such improvement in inhibitory potency is inconsistent with C37 and 5-Helix binding to completely independent states during the fusion process. Instead, C37 appears to interact more efficiently with 5-Helix-bound gp41, perhaps because the N-HR coiled coil is exposed for longer duration.

Curiously, when this experiment was carried out in reverse and fusion was first trapped in the C37_W628A_-bound intermediate state, the potency of 5-Helix inhibition showed comparatively little enhancement (<2-fold, Figure 6B, Figure S6B). Thus, in contrast with C37 binding to 5-Helix-trapped gp41, there appears to be no substantial improvement in 5-Helix binding to the C37-trapped state. This qualitative difference in trapped conformations suggests that the gp41 N-HR and C-HR regions are not exposed symmetrically in a single state during membrane fusion.

Asymmetric exposure of the gp41 N-HR and C-HR may also explain the combined antiviral activity of C37 and 5-Helix. Since wild type C37 and 5-Helix tightly associate at nanomolar concentrations (K_D_ = 0.65 pM), these two inhibitors are highly antagonistic when used together [3],[16], making them inappropriate for combinatorial studies. Instead, we utilized C37_N656D_ and 5-Helix_V549E_ because their binding affinity is relatively weak (K_D_ = 160 nM) compared to their individual inhibitory potencies (see legend to Figure 6). With both inhibitors at low concentrations, we observed additive antiviral activity, as if the inhibitors targeted separate intermediate states (modeled by the solid line in Figure 6C; for details, see Text S1 and Figure S7). At high concentrations, the combined inhibitory activity showed considerable synergy, as if the inhibitors bound simultaneously to a single gp41 intermediate state (modeled by the dotted line in Figure 6C). The gradual shift from additive to synergistic activity (Figure 6D) suggests that neither model alone perfectly describes the inhibition process. Rather, the data point to multiple prehairpin intermediate conformations, some that exclusively bind C37 or 5-Helix and others that can bind both inhibitors simultaneously.

## Discussion

The growing class of intermediate-state inhibitors includes antibiotics, immunosuppressive agents, and anesthetics used in the research and clinical settings [1],[2],[29],[30]. Also known as uncompetitive or use-dependent inhibitors, these agents bind transiently accessible targets, and, accordingly, their potencies are not simple functions of equilibrium binding affinity [31]. Kinetic parameters such as the lifetime of the sensitive state and the rate of inhibitor association can strongly influence the level of inhibition [3]. Here, we have shown that the final disposition of the inhibitor-bound state also critically affects the activity of intermediate state inhibitors.

Inhibitor binding to the gp41 prehairpin intermediate state promotes the irreversible deactivation of HIV-1 membrane fusion. For C37-bound gp41, deactivation is a slow process, and most C37 variants have time to dissociate to some degree. Due to the reversibility of inhibition, C37 potency depends upon binding affinity for all but the tightest binding variants. Similar affinity dependence has been observed for the potencies of other N-HR-targeting inhibitors, including antibodies [25],[32], D-peptides [24] and other C-peptides [11],[33]. This correlation suggests that slow deactivation is a general property for inhibitors that target the gp41 N-HR region. Conversely, 5-Helix-bound gp41 rapidly deactivates before most 5-Helix variants have time to dissociate. Consequently, 5-Helix inhibition is functionally irreversible, and potency depends primarily on the rate of inhibitor association. It remains to be seen whether rapid deactivation is a general property for other engineered proteins and antibodies that target the C-terminal region of the gp41 ectodomain.

C37-bound gp41 and 5-Helix-bound gp41 appear to deactivate through distinct mechanisms that differ in both their time course and chemokine-receptor dependence. For C37 inhibition, the transition rate out of the unbound intermediate state and the deactivation rate of the bound state (k_f_ and k_s-C37_) are equally sensitive to chemokine-receptor levels. Thus, chemokine-receptor binding to gp120 appears to limit the lifetimes of both the unbound and C37-bound states. The event likely alters the association of gp120 and gp41, triggering TOH formation when the N-HR is unbound, but causing gp41 to misfire when the N-HR is bound. By contrast, the rapid deactivation rate of 5-Helix-bound gp41 is independent of chemokine-receptor levels. Perhaps 5-Helix binding directly induces gp41 misfolding, possibly by altering the manner by which the C-terminal region of the gp41 ectodomain interacts with gp120 or the viral membrane. Alternatively, during the natural structural progression of the prehairpin intermediate state, Env conformations might arise that sterically block 5-Helix from dissociating, irreversibly trapping the inhibitor on gp41. Whatever the mechanism, rapid, chemokine-receptor independent deactivation may represent an advantageous property to engineer into future HIV-1 membrane fusion inhibitors.

The success of T20 (enfuvirtide) in the clinic has spurred considerable efforts to design improved C-peptide inhibitors of HIV-1 entry [33]–[37]. C-peptide variants have been engineered to interact more strongly with the N-HR coiled coil, but none are significantly more potent against wild type HIV-1 than the original peptides. We suspect that these tighter binding variants are kinetically restricted inhibitors much like C37-KYI and di-C37. Their inhibition is effectively irreversible, and, consequently, their potencies depend only on the lifetime of N-HR exposure and the rate of inhibitor association. Interestingly, the potency of C-peptide C34 against wild type HIV-1 strains is substantially improved (15 to 50-fold) when a cholesterol moiety is added specifically at the peptide C-terminus [38]. Rather than increasing binding affinity, the modification concentrates the peptide on target cell membranes, optimally prepositioning the inhibitor to bind gp41 rapidly following N-HR exposure. Hence, C-peptide potency can be improved by increasing the rate of inhibitor association.

Although affinity enhancement does not improve their potency against wild type virus, tighter binding C37 variants do represent improved gp41 inhibitors. Escape from C-peptide inhibition occurs largely through mutations in the N-HR segment that directly disrupt peptide affinity [26]–[28],[39],[40]. The extra binding strength of kinetically restricted inhibitors acts as a “resistance capacitor” [24], enabling these peptides to retain their potency in the setting of affinity-reducing escape mutations [28], [34]–[36]. Consistently, we have found that resistance to C37-KYI and di-C37 takes much longer to achieve and requires more escape mutations than resistance to wild type C37 (KMK and MJR, manuscript in preparation). Similar genetic barriers to resistance have been reported for other second generation C-peptide inhibitors [28],[34],[41].

Our synergy data suggest that C37 and 5-Helix can bind the same gp41 intermediate, even though they promote Env deactivation through different mechanisms. This prehairpin state, however, probably does not adopt a single, static conformation with the N-HR and C-HR regions symmetrically exposed. Previous studies of lipid- and temperature-arrested Env suggest that the intermediate conformation evolves, with the N-HR becoming progressively exposed and the C-HR becoming progressively occluded [20]. These conclusions informed our interpretation of the synergy data that C37 and 5-Helix target partially, but not completely, overlapping states. What, then, might account for this asymmetry in N-HR and C-HR exposure? A possible candidate is gp120. A recent study of Env subunit association suggests that CD4-bound gp120 can interact with gp41-derived peptides containing the C-HR sequence [42]. A similar association is not observed with N-HR-derived peptides. Based on this result, we propose that an interaction between gp120 and the C-HR limits exposure of this gp41 segment after Env activation. The presence of a bound 5-Helix would disrupt the gp120/C-HR interaction, leading to chemokine receptor-independent Env deactivation. By contrast, the N-HR coiled coil, once formed, remains accessible to gp41 inhibitors until TOH formation. With no competing interactions, the N-HR would potentially tolerate a bound C-peptide until chemokine receptor triggered the final gp41 conformational changes.

## Materials and Methods

### Cell Lines

The following reagents were obtained through the AIDS Research and Reference Reagent Program, Division of AIDS, NIAID, NIH: HOS-CD4-Fusin and HOS-CD4-CCR5 from Dr. Nathaniel Landau [43]; HeLa-CD4-LTR-β-gal from Dr. Michael Emerman [44]. In addition, RC30 and RC49 were kindly provided by Dr. David Kabat (Oregon Health Sciences University) [45].

### Peptide and Protein Production

C37 is a His-tagged C-peptide derived from HIV-1_HXB2_ Env residues 625–661 (Figure 4A) [16]. Wild type and mutant peptides were generated through proteolysis of the recombinantly produced trimer-of-hairpins construct NC1. This protein was expressed in *E.coli* and purified from bacterial lysates using Ni-NTA Agarose (Qiagen) per manufacturer's protocol. Eluted NC1 was incubated with trypsin (Sigma, 1∶250 mass ratio) overnight at 4°C. The resulting C37 was purified to homogeneity by reverse phase HPLC (Vydac C18 column) using a water:acetonitrile gradient in trifluoroacetic acid (0.1%). The identity of all C37 peptides was confirmed by mass spectrometry.

5-Helix is a 25 kD His-tagged protein consisting of three N-HR segments (Env_HXB2_ residues 542–581) and two C-HR segments (residues 625–662) alternately connected into a single polypeptide [16]. This protein was recombinantly expressed in E.coli and solubilized from bacterial inclusion bodies using 8 M guanidine HCl (GdnHCl) in tris-buffered saline (TBS) [3]. Following initial purification using Ni-NTA agarose, 5-Helix-bound beads were heated to 90°C in 4 M GdnHCl and allowed to cool to room temperature overnight to promote protein refolding. 5-Helix was eluted with imidazole in TBS, and monomers were purified from aggregates on a Sephacryl S200 HR column (GE). The concentrations of all C37 and 5-Helix polypeptides were determined by absorbance at 280 nm by the method of Edelhoch [46].

For interaction experiments, cysteinated versions of C37 and 5-Helix were labeled with rhodamine- or fluorescein-maleimide (Molecular Probes) [3]. C37 with an N-terminal Cys was labeled prior to HPLC purification. 5-Helix with a C-terminal Cys was labeled on beads under denaturing conditions prior to refolding. The concentrations of these fluorescent polypeptides were determined by absorbance using extinction coefficients of 87,500 M^−1^ cm^−1^ at 490 nm for fluorescein (in potassium phosphate pH 9) and 95,000 M^−1^ cm^−1^ at 520 nm for rhodamine (in methanol). These concentrations were verified through stoichiometric titrations using unlabeled C37 and 5-Helix of known concentrations.

### Interaction Measurements

Reported K_D_ and k_on_ values were measured for the solution-phase interaction of cognate-binding partners C37 and 5-Helix. This interaction models C37 and 5-Helix binding to the gp41 N-HR and C-HR, respectively. All experiments were carried out at 25°C in TBS supplemented with 100 µg/ml BSA, 0.02% NaN_3_, and 1 mM PMSF. To determine C37 binding parameters, the peptide was titrated into a fixed concentration of fluorescein-labeled 5-Helix and incubated for 2.9 seconds for kinetic measurements or for up to 72 hours for equilibrium measurements. The concentration of unbound 5-Helix was determined using a KinExA 3000 flow fluorimeter (Sapidyne Instruments) with azlactone beads (Pierce) covalently coupled to C37 per manufacturer's protocol. 5-Helix captured by these beads led to a change in bead fluorescence that was proportional to the unbound 5-Helix concentration in solution (Figure S1). The C37 concentration dependence of the fluorescence signal was fit to a general model of bimolecular interactions using manufacturer's software. 5-Helix binding parameters were determined using similar methods except that i) 5-Helix was titrated into a fixed concentration of rhodamine-labeled C37, and ii) the beads were covalently coupled to 5-Helix. Dissociation constants (k_off_) were calculated using the equation k_off_ = K_D_•k_on_. Details of these binding assays can be found in reference [3].

### Viral Inhibition Assay

C37 and 5-Helix inhibitory potencies were determined using single-round viral infectivity assays as previously described [11]. Briefly, virions pseudotyped with Env_HXB2_, Env_JR-FL_ or Env_Ba-L_ were generated by cotransfection of the Env-deficient HIV-1_NL4-3_ genome (pNL4-3R^-^E^-^Luc^+^ [47]) and an Env-expressing plasmid (pEBB_Env) into 293T cells. HIV-1 harvested 48 hours post-transfection was used to infect appropriate target cells (see below) in the presence of varying inhibitor concentrations. The level of viral infectivity was measured 48 hours later by assaying for luciferase production in infected cells (Luciferase Assay System, Promega). Data were fit to a Langmuir equation to obtain IC_50_ values (see Figure S1A).

Target cells expressed CD4 and varying concentrations of CXCR4 (for Env_HXB2_) or CCR5 (for Env_JR-FL_ and Env_Ba-L_). For HIV-1_HXB2_ infections, we utilized HOS-CD4-CXCR4 (high CXCR4) or HOS-CD4-CCR5 (low CXCR4) cells, which express CXCR4 at levels of approximately 10^5^ and 5×10^3^ molecules per cell, respectively (HKS and MJR, unpublished results). For HIV-1_Ba-L_ infections, we utilized RC49 and RC30 cells, which express CCR5 at levels of 8.5×10^4^ and 2.4×10^3^ molecules per cell, respectively [45]. By comparison, the typical range of endogenous expression is 10^3^ to 10^4^ CXCR4 molecules per cell (PBMCs [48]) and 2×10^4^ CCR5 molecules per cell (activated CD4+ human T-cells [45]). For HIV_JR-FL_ infections, we utilized HOS-CD4-CCR5 cells.

### Inhibitor-Washout Viral Infectivity Assay

These experiments were designed to measure the reversibility of C37 and 5-Helix inhibition. Care was taken to strictly maintain a temperature of 37°C for all cellular washes and incubation steps. HeLa-CD4-LTR-β-gal target cells were seeded in a 96-well plate at 1.6×10^4^ cells/well. The following day, these cells were preincubated with HIV-1_NL4-3_ at 37°C in the presence of >IC95 concentrations of C37 or 5-Helix. After 2 hours, cells were rapidly and thoroughly washed (3×100 µl, see Figure S3) with warm media containing no gp41 inhibitor and 100 µg/ml of anti-CD4 antibody #19 (J. Hoxie, University of Pennsylvania) to prevent activation of any unengaged Env. These washout samples were incubated for varying times (0–240 minutes) to permit inhibitor-bound Env to recover its fusion activity. Additional preincubated samples were washed and incubated in 1 µM C37 in order to measure the small level of background infection that occurred during the 2-hour preincubation phase. Infections were terminated upon the addition of 1 µM C37, and cells were maintained another 24 hours to enable reporter expression. Cell lysates (100 mM potassium phosphate, 100 mM sodium phosphate, 0.1% triton, pH 7) were assayed for β-galactosidase expression using Lumi-Gal 530 (Lumigen, Inc.). The difference between the washout infection level and background infection level reflects fusion recovery due to inhibitor dissociation. Recovery fraction was calculated by normalizing this difference to the infection level obtained when HIV-1 was preincubated in the absence of inhibitor.

The assay was slightly modified to assess C37 and 5-Helix potency against inhibitor-bound gp41. Following preincubation with C37_W628A_ or 5-Helix_L556A/V570A_, cells were washed with media that contained the complementary inhibitor and incubated 1 hour. Infections were terminated and subsequently analyzed as described above. The dependence of recovery fraction on inhibitor concentration was compared to standard titrations of HIV-1_NL4-3_ infectivity (with virus and inhibitor coincubated for the duration of infection).

## Supporting Information

Table S1 Inhibitory and binding properties of 5-Helix variants
(0.09 MB PDF)

Table S2 Inhibitory and binding properties of C37 variants
(0.10 MB PDF)

Text S1 Modeling the inhibitory activities of C37 and 5-Helix combinations
(0.05 MB PDF)

Figure S1 Binding and inhibitory properties of selected C37 variants. (A) Inhibition of HIV-1 infectivity by C37 (black) and two lower affinity variants, W628A (red) and L645D (green). Data are representative of a single experiment and reflect the mean ± ROM of duplicate measurements. Solid lines represent a fit of the data to a Langmuir equation to obtain IC50 values. (B) KinExA 3000 fluorescence response to equilibrated solutions of 30 pM 5-Helix-fluorescein and the shown concentrations of C37_W628A_. The KinExA instrument was configured to capture a portion of unbound 5-Helix within its flow cell in order to determine the free 5-Helix concentration in solution. The arrows labeled I and W represent sample injection and buffer wash. (C) Titration of 30 pM 5-Helix-fluorescein (5H-F) by C37 (black), C37_W628A_ (red) and C37_L645D_ (green). Data have been fit to a general bimolecular equilibrium binding model to determine K_D_ values. (D) KinExA 3000 fluorescence response to pre-equilibrated solutions of 1 nM 5-Helix-fluorescein and various concentrations of C37_W628A_. Solutions were mixed for 2.9 seconds prior to passage through the instrument flow cell. (E) Nonequilibrium titration of 1 nM 5-Helix-fluorescein by C37 (black), C37_W628A_ (red) and C37_L645D_ (green). Data have been fit to a kinetic bimolecular binding model to determine k_on_ values.
(0.38 MB PDF)

Figure S2 Affinity and kinetic dependence to antiviral potency. IC50 values for the 5-Helix (A) and C37 (B) variants are plotted as a function of both K_D_ and k_on_. The data are color coded as in Figure 1 and globally fit to Equation 1 (blue mesh). The estimated k_f_ and k_s_ values are: for C37, k_f_ = 0.054 sec^−1^, k_s-C37_ = 0.00049 sec^−1^; for 5-Helix, k_f_ = 0.21 sec^−1^, k_s-5H_ = 0.11 sec^−1^.
(0.80 MB PDF)

Figure S3 Assessment of inhibitor washout efficiency. Target cells were incubated for 2 hours with high concentrations (>IC95) of C37 variants (A) or 5-Helix variants (B) used in the inhibitor-washout viral infectivity assay. The washout procedure (3×100 µl media) was performed and cells were subsequently infected with HIV-1_NL4-3_ overnight. Measured viral infectivity was normalized to a no-inhibitor control. Mean values ± SEM for three independent experiments are shown.
(0.20 MB PDF)

Figure S4 Recovery of gp41 fusion activity from PIE7 inhibition. PIE7 is a short, rigid peptide composed of D-amino acids that targets the deep hydrophobic pocket of the N-HR coiled coil [24]. A crosslinked dimer of PIE7 inhibits HIV-1 entry more potently than the monomeric form, presumably due to the enhanced binding strength afforded by multivalent interactions. The timecourse of fusion recovery from PIE7 (black) and PIE7-dimer (red) blockade was measured as described in Figure 3, except that the virus used was HIV-1_HXB2_ and the target cells were HOS-CD4-CXCR4. The points represent the mean ± ROM of two independent experiments.
(0.18 MB PDF)

Figure S5 Effect of CCR5 levels on 5-Helix- and C37-inhibitory activity against HIV-1_Ba-L_. IC50 values were determined utilizing RC49 (black) and RC30 (gray) target cells expressing high and low levels of CCR5, respectively (see Materials and Methods). Inhibitors are ordered according to increasing k_off_ values as measured for HXB2 sequences. The data represent the mean ± SEM of three independent experiments.
(0.19 MB PDF)

Figure S6 Sensitivity of inhibitor-trapped gp41 to wild type C37 and 5-Helix. (A) C37 inhibitory activity was measured by standard assay (squares) or in a 5-Helix-washout assay after Env was first trapped in the 5-Helix_L556A/V570A_-bound state (circles). (B) 5-Helix inhibitory activity was measured by standard assay (squares) or in a C37-washout assay after Env was first trapped in the C37_W628A_-bound state (circles). Experiments were conducted as described in the legend to Figure 5.
(0.18 MB PDF)

Figure S7 Simulating the combined inhibitory activities of 5-Helix and C37. (A, B, C) Models of intermediate state inhibition by two different inhibitors X and Y. In Model 1 (A), the inhibitors bind separate states. In Model 2 (B), the inhibitors bind separately to the same state. In Model 3 (C), the inhibitors can bind simultaneously to the same state. (D, E, F) Monte Carlo simulation of the inhibitory activities of 5-Helix_V549E_ and C37_N656D_ alone (symbols). The solid lines correspond to the expected titrations based upon the respective IC50 values (54 nM for 5-Helix_V549E_; 130 nM for C37_N656D_) and calculated using a Langmuir function (Equation S1, see Text S1). (G, H, I) Monte Carlo simulation of the C37_N656D_ inhibitory activity in the presence of 30 nM 5-Helix_V549E_. Solid lines correspond to the analytical solution of fusion probability for Models 1 and 2 (Equations S2 and S3). Simulated points represent the average of 10^5^ iterations. The interaction between 5-Helix_V549E_ and C37_N656D_ (K_D_ = 165 nM) was taken into account for all simulations and calculations. Details of the simulation procedure and derivation of the analytical formulas are presented in Text S1.
(0.25 MB PDF)
