# Supplementary material for: Asymmetric Deactivation of HIV-1 gp41 following Fusion Inhibitor Binding
Source: PLoS Pathog. 2009 Nov 26;5(11):e1000674. doi: 10.1371/journal.ppat.1000674 (PMC2776349; doi:10.1371/journal.ppat.1000674)
Supplement: Table S1 — Inhibitory and binding properties of 5-Helix variants (0.09 MB PDF) [file ppat.1000674.s001.pdf]

Table S1. Inhibitory and Binding Properties of 5-Helix Variants.

5-Helix Variant Class	Variant[a]	3x or N3[b]	K_D ± SEM (nM)[c]	k_{on} ± SEM ($x10^7 M^{-1}s^{-1}$)[c]	k_{off} ± SEM (s^{-1})[d]	τ_{off} (s)[e]	IC_{50} ± SEM (nM)[f]
WT ■[g]	WT	N/A	0.00065±0.0002	3.3±0.43	0.000021±0.0000035	47000	11±1.2
Single Asp ■	Q563D	3x	0.049±0.0061	1.0±0.1	0.0005±0.000092	2000	38±3
	V570D	3x	2.3±0.39	0.35±0.07	0.0081±0.002	120	90±9
Double Ala ■	L556A/V570A	3x	8.3±0.95	1.2±0.22	0.083±0.021	12	17±2.4
Ala+Asp ■	V549D/L556A	N3	40±7.3	0.028±0.005	0.011±0.0029	91	2400±62
	L556A/Q563D	N3	14±4.4	1.1±0.22	0.15±0.057	6.7	44±6.9
	L556A/V570D	N3	74±14	0.033±0.002	0.024±0.0049	42	1600±420
Double Asp ■	V549D/Q563D	N3	25±4.5	0.21±0.04	0.053±0.014	19	140±37
	L556D/Q563D	N3	250±73	0.043±0.001	0.11±0.031	9.1	860±150
	Q563D/V570D	N3	8±0.84	0.13±0.01	0.01±0.0015	100	380±53
Triple Ala ■	L556A/Q563A/V570A	3x	29±4.5	2.6±0.5	0.75±0.12	1.3	35±1
	V549A/L556A/Q563A	3x	35±1	>2.0	>0.7	<1.4	16±1
	V549A/Q563A/V570A	3x	9.2±0.55	0.62±0.085	0.055±0.0084	18	34±2

[a]Residue numbering according to Env_{HXB2} sequence.

[b]Distinguishes whether substitutions were incorporated into all three N-HR segments (3x) or incorporated in the third N-HR segment only (N3). For details, see Steger and Root (2006) J Biol Chem 281: 25813-21.

[c]Solution phase interaction parameters determined using KinExA 3000 binding assay (mean ± SEM of three or more independent experiments).

[d]Values for k_{off} were calculated from K_D and k_{on} measurements ($k_{off} = K_D k_{on}$) with errors formally propagated.

[e]The dissociation time constant (τ_{off}) is the reciprocal of the k_{off} value.

[f]Inhibitory potencies determined in single-round $HIV\text{-}1_{HXB2}$ infectivity assays using HOS-CD4-CXCR4 target cells (mean ± SEM of three or more independent experiments).

[g]Symbols utilized in Figs. 1B, 1D and S2A.
