# Supplementary material for: Asymmetric Deactivation of HIV-1 gp41 following Fusion Inhibitor Binding
Source: PLoS Pathog. 2009 Nov 26;5(11):e1000674. doi: 10.1371/journal.ppat.1000674 (PMC2776349; doi:10.1371/journal.ppat.1000674)
Supplement: Table S2 — Inhibitory and binding properties of C37 variants (0.10 MB PDF) [file ppat.1000674.s002.pdf]

Table S2. Inhibitory and Binding Properties of C37 Variants.

C37 Variant Class	Variant[a]	Mutation Position[b]	K_D ± SEM (nM)[c]	k_{on} ± SEM ($x10^7 M^{-1}s^{-1}$)[c]	k_{off} ± SEM (s^{-1})[d]	τ_{off} (min)[e]	IC_{50} ± SEM (nM)[f]
WT ■[g]	WT	-	0.00065±0.000063	3.3±0.43	0.000021±0.0000035	780	1.25±0.14
Ala ■	W628A	A	0.033±0.004	1.5±0.021	0.0005±0.000095	33	16±2.2
	W631A	D	1.7±0.56	1.3±0.1	0.022±0.0075	0.75	110±9.7
	I635A	A	0.0066±0.0005	2.1±0.3	0.00014±0.000023	120	5.7±1.6
	Y638A	D	0.001	2.5	0.000025	670	2.5±0.33
	I642A	A	0.0036±0.001	1.9±0.12	0.000068±0.00002	250	7.0±1.5
	L645A	D	0.013±0.0057	2.4±0.1	0.00031±0.00014	54	3.9±0.7
	S649A	A	0.00071±0.00003	2.5±0.39	0.000018±0.0000028	930	1.6±0.41
	Q652A	D	0.0029±0.0003	3.4±0.42	0.000096±0.000015	170	3.5±0.81
	N656A	A	0.0045±0.0009	1.5±0.17	0.000069±0.000016	240	30±4.2
	E659A	D	0.00063	3.6±0.3	0.000023	720	1.2±0.13
Asp ■	W628D	A	0.027±0.0067	1.3±0.053	0.00033±0.000086	50	7.6±1.0
	W631D	D	2.7±0.53	1.0±0.11	0.028±0.0062	0.60	550±65
	I635D	A	14±2.4	2.5±0.15	0.34±0.062	0.049	3100±350
	Y638D	D	0.20±0.015	0.42±0.032	0.00083±0.000091	20	39±0.92
	I642D	A	62±1.7	-	-	-	13000±2100
	L645D	D	3.2±0.65	1.4±0.083	0.045±0.0094	0.37	630±120
	S649D	A	3.8±0.58	1.8±0.3	0.067±0.015	0.24	2300±170
	Q652D	D	0.027±0.0059	1.6±0.19	0.00043±0.00011	39	53±0.98
	N656D	A	2.5±0.75	2.6±0.058	0.065±0.020	0.26	100±8.9
Asp+Ala ■	W628D/I635A	A/A	1.0±0.14	0.47±0.042	0.0048±0.00077	3.5	97 ±8.4
	W628D/I642A	A/A	0.30±0.053	0.49±0.035	0.0015±0.00028	11	65±9.8
	W631D/I642A	D/A	321±50	0.10±0.022	0.32±0.086	0.052	15000±2700
Kinetically Restricted Variants	N637K (KYT)	C	0.00013±0.000036	2.3±0.016	0.000003±0.000021	5600	1.65±0.05
	T639I (NYI)	E	0.000080±0.000018	2.3±0.016	0.0000018±0.000013	9300	1.7±0.1
	N637K/T639I (KYI)	C/E	0.000062±0.000016	2.4±0.1	0.0000015±0.000062	11000	0.96±0.1
	di-C37	-	-	-	-	-	0.7±0.26

[a]Residue numbering according to Env_{HXB2} sequence.
[b]Residue position in helical wheel diagram according to Chan, et al (1997) Cell 89: 263-73.
[c]Solution phase interaction parameters determined using KinExA 3000 binding assay (mean ± SEM of three or more independent experiments).
[d]Values for k_{off} were calculated from K_D and k_{on} measurements ($k_{off} = K_D k_{on}$) with errors formally propagated.
[e]The time constant for dissociation (τ_{off}) is the reciprocal of the k_{off} value.
[f]Inhibitory potencies determined in single-round HIV-1_{HXB2} infectivity assays using HOS-CD4-CXCR4 target cells (mean ± SEM of three or more independent experiments).
[g]Symbols utilized in Figs. 1C, 1E and S2B.
