# Supplementary material for: Asymmetric Deactivation of HIV-1 gp41 following Fusion Inhibitor Binding
Source: PLoS Pathog. 2009 Nov 26;5(11):e1000674. doi: 10.1371/journal.ppat.1000674 (PMC2776349; doi:10.1371/journal.ppat.1000674)
Supplement: Text S1 — Modeling the inhibitory activities of C37 and 5-Helix combinations (0.05 MB PDF) [file ppat.1000674.s003.pdf]

Supporting Information

Modeling the inhibitory activities of C37 and 5-Helix combinations

To understand the combined antiviral activity of C37 and 5-Helix, we explored three inhibition models that differed in the way two inhibitors could bind gp41 intermediate states (Figure S7). In the first model, C37 and 5-Helix (arbitrarily designated as X and Y) bind independently to two separate intermediate conformations (I_1 and I_2). In the second model, the inhibitors target the same intermediate state (I), but their binding is mutually exclusive (i.e., only one inhibitor can bind at a time). In the third model, the inhibitors can bind simultaneously to the same intermediate conformation. In all cases, inhibitor binding leads to irreversible gp41 deactivation. The rate constants in each scheme are defined as follows:

k_{f1}	Rate out of the X-sensitive state	k_{f2}	Rate out of the Y-sensitive state
k_1	Association rate constant for X	k_2	Association rate constant for Y
k_{-1}	Dissociation rate constant for X	k_{-2}	Dissociation rate constant for Y
k_{sx}	Deactivation rate of I-X	k_{sy}	Deactivation rate of I-Y
k_{sxy}	Deactivation rate of I-XY (only for model 3)		

In the presence of only one inhibitor (Inh), the probability that the system will end up in the fusogenic conformation (P_F) is related to inhibitor concentration ([Inh]) by the Langmuir function:

$$P_F = \frac{1}{1 + \frac{[\mathrm{Inh}]}{\mathrm{IC50}_{\mathrm{Inh}}}} \qquad \text{(S1)}$$

$\mathrm{IC50}_{\mathrm{Inh}}$ formulas for X and Y can be derived in terms of the defined rate constants (see reference [1] for derivation):

$$\mathrm{IC50_X} = \frac{k_{f1}}{k_1}\left(1+\frac{k_{-1}}{k_{sx}}\right) \qquad \mathrm{IC50_Y} = \frac{k_{f2}}{k_2}\left(1+\frac{k_{-2}}{k_{sy}}\right) \tag{S2}$$

In models 1 and 2, X and Y bind separately, and, thus, their combined inhibition should be additive. Fusion probabilities can be easily derived in terms of $\mathrm{IC50_X}$ and $\mathrm{IC50_Y}$ (Equation S2) for both models (see appendix):

(Model 1)
$$P_\mathrm{F} = \frac{1}{1+\frac{[\mathrm{X}]}{\mathrm{IC50_X}}+\frac{[\mathrm{Y}]}{\mathrm{IC50_Y}}+\frac{[\mathrm{X}][\mathrm{Y}]}{\mathrm{IC50_X}\cdot\mathrm{IC50_Y}}} \tag{S3}$$

(Model 2)
$$P_\mathrm{F} = \frac{1}{1+\frac{[\mathrm{X}]}{\mathrm{IC50_X}}+\frac{[\mathrm{Y}]}{\mathrm{IC50_Y}}} \tag{S4}$$

These formulas have been previously derived for additive inhibition of mutually non-exclusive (Equation S3) and mutually exclusive (Equation S4) inhibitors [2].

For model 3, the simultaneous binding X and Y could enable synergistic inhibitory activity (the binding of one inhibitor enhances the inhibitory activity of the other). Unfortunately, the fusion probability for this model does not have a simple analytical solution. Therefore, we performed Monte Carlo “random walk” simulations to determine the concentration dependence to fusion probabilities. Simulations were implemented in Visual Basic run through an Excel spreadsheet (Microsoft). Transition probabilities were assigned according to the rates out of each state. For example, the transition probabilities out of the I and I-XY states (see Figure S7C) are:

$$\begin{aligned}
P_{\mathrm{I}\to\mathrm{IX}} &= \frac{k_1[\mathrm{X}]}{k_1[\mathrm{X}]+\gamma k_2[\mathrm{Y}]+k_{f1}} & P_{\mathrm{IXY}\to\mathrm{IX}} &= \frac{k_{-2}}{k_{-1}+k_{-2}+k_{sxy}} \\
P_{\mathrm{I}\to\mathrm{IY}} &= \frac{\gamma k_2[\mathrm{Y}]}{k_1[\mathrm{X}]+\gamma k_2[\mathrm{Y}]+k_{f1}} & P_{\mathrm{IXY}\to\mathrm{IY}} &= \frac{k_{-1}}{k_{-1}+k_{-2}+k_{sxy}} \\
P_{\mathrm{I}\to\mathrm{F}} &= \frac{k_{f1}}{k_1[\mathrm{X}]+\gamma k_2[\mathrm{Y}]+k_{f1}} & P_{\mathrm{IXY}\to\mathrm{DXY}} &= \frac{k_{sxy}}{k_{-1}+k_{-2}+k_{sxy}}
\end{aligned} \tag{S5}$$

(The parameter γ equals k_{f2}/k_{f1} and accounts for the small difference in the lifetimes of the X- and Y-sensitive states.) For each [X] and [Y], 10^4 to 10^6 random walks were performed, with each iteration being initiated in state I and terminated in state F, D-X, D-Y or D-XY. The fraction of iterations ending in state F was assigned P_F. The simulation was applied to the inhibition of $C37_{N656D}$ and 5-$Helix_{V549E}$ combinations using rate constants determined in this study:

X = $C37_{N656D}$		Y = 5-$Helix_{V549E}$	
k_{f1}	0.05 sec^{-1}	k_{f2}	0.2 sec^{-1}
k_1	2.6 x 10^7 $M^{-1}sec^{-1}$	k_2	0.6 x 10^7 $M^{-1}sec^{-1}$
k_{-1}	0.04 sec^{-1}	k_{-2}	0.05 sec^{-1}
k_{sx}	4.9 x 10^{-4} sec^{-1}	k_{sy}	0.11 sec^{-1}
k_{sxy}	0.11 sec^{-1}		

The rate constant k_{sxy} was assumed to be equivalent to $k_{s\text{-}5H}$ on the basis of the following observations: i) in inhibitor-washout viral infectivity studies performed in the presence of both C37 and 5-Helix variants, the 5-Helix variant phenotype was dominant (the levels of recovery when both inhibitors were present and when 5-Helix alone was present were equal). ii) The simulations do not show synergistic activity when k_{sxy} is equivalent to $k_{s\text{-}C37}$.

To verify the accuracy of this Monte Carlo method, simulations were performed for models 1 and 2 and compared to the analytical inhibition solutions (Equations S3 and S4). Additional simulations were performed with only one inhibitor present. The data are presented in Figure S7.

Appendix: Derivation of additive inhibition formulas

Models 1 and 2 can be rewritten in the following simplified forms:

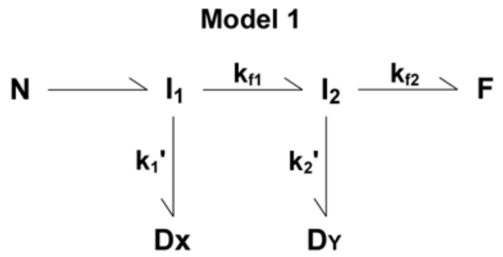

Here, the binding and deactivation steps are represented as a net reaction with a unidirectional rate constant k' (as derived in Steger and Root, reference [1]):

$$k_1' = \frac{k_{sx}k_1[\mathrm{X}]}{k_{-1} + k_{sx}} \qquad k_2' = \frac{k_{sy}k_2[\mathrm{Y}]}{k_{-2} + k_{sy}} \qquad \text{(S6)}$$

The k' expressions can be rewritten in terms of $\mathrm{IC50_X}$ and $\mathrm{IC50_Y}$ using Equation S2:

$$k_1' = \frac{k_{f1}[\mathrm{X}]}{\mathrm{IC50_X}} \qquad k_2' = \frac{k_{f2}[\mathrm{Y}]}{\mathrm{IC50_Y}} \qquad \text{(S7)}$$

For Model 1, the probability of getting through to state F is equal to the probability of transitioning from I_2 to F given that the transition from I_1 to I_2 had already occurred ($P_{\mathrm{I_1 \to F}} = P_{\mathrm{I_1 \to I_2}} \cap P_{\mathrm{I_2 \to F}}$). P_F can be evaluated as follows:

$$\text{(Model 1)} \qquad P_{\mathrm{F}} = \left(\frac{k_{f1}}{k_{f1} + k_1'}\right)\left(\frac{k_{f2}}{k_{f2} + k_2'}\right) = \frac{1}{1 + \frac{k_1'}{k_{f1}} + \frac{k_2'}{k_{f2}} + \frac{k_1'}{k_{f1}}\frac{k_2'}{k_{f2}}} \qquad \text{(S8)}$$

Replacing k' in Equation S8 with the expressions in Equation S7 brings the P_F formula for Model 1 into the form of Equation S3.

For Model 2, the fusion probability is merely

$$\text{(Model 2)} \qquad P_{\mathrm{F}} = \left(\frac{k_f}{k_f + k_1' + k_2'}\right) \qquad \text{(S9)}$$

Dividing through by k_f and substituting for k' brings Equation S9 into form for P_F shown in Equation S4.

References

1. Steger HK, Root MJ (2006) Kinetic dependence to HIV-1 entry inhibition. J Biol Chem 281**:** 25813-21.

2. Chou TC, Talalay P (1984) Quantitative analysis of dose-effect relationships: the combined effects of multiple drugs or enzyme inhibitors. Adv Enzyme Regul 22**:** 27-55.
