# Supplementary figures and images for: Asymmetric Deactivation of HIV-1 gp41 following Fusion Inhibitor Binding
Source: PLoS Pathog. 2009 Nov 26;5(11):e1000674. doi: 10.1371/journal.ppat.1000674 (PMC2776349; doi:10.1371/journal.ppat.1000674)

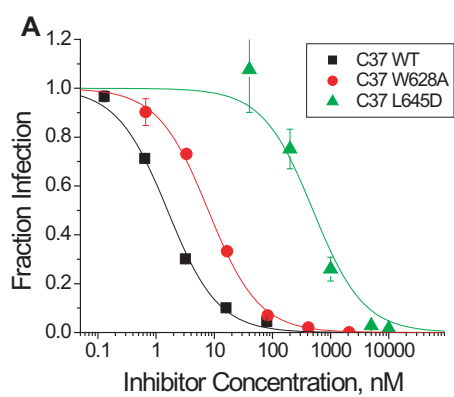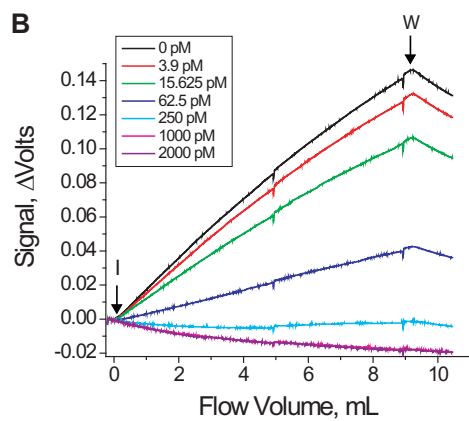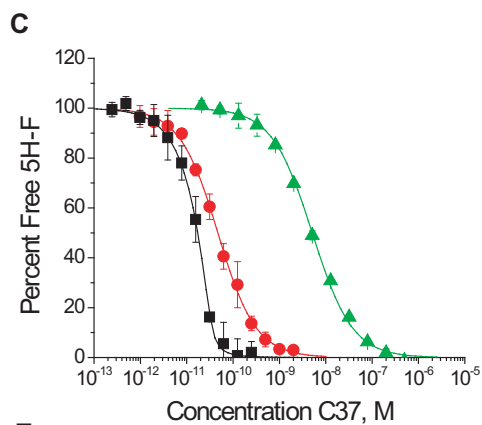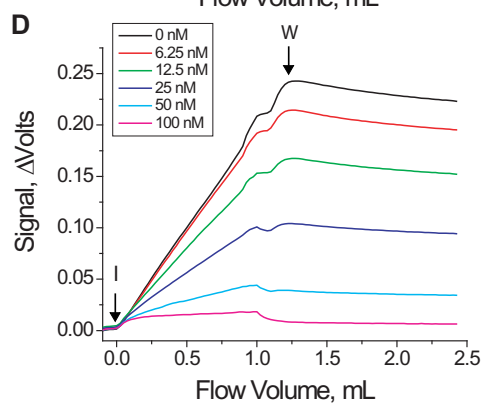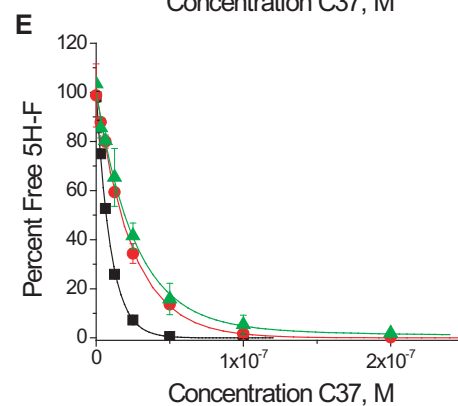

Supplement: Figure S1 — Binding and inhibitory properties of selected C37 variants. (A) Inhibition of HIV-1 infectivity by C37 (black) and two lower affinity variants, W628A (red) and L645D (green). Data are representative of a single experiment and reflect the mean ± ROM of duplicate measurements. Solid lines represent a fit of the data to a Langmuir equation to obtain IC50 values. (B) KinExA 3000 fluorescence response to equilibrated solutions of 30 pM 5-Helix-fluorescein and the shown concentrations of C37W628A. The KinExA instrument was configured to capture a portion of unbound 5-Helix within its flow cell in order to determine the free 5-Helix concentration in solution. The arrows labeled I and W represent sample injection and buffer wash. (C) Titration of 30 pM 5-Helix-fluorescein (5H-F) by C37 (black), C37W628A (red) and C37L645D (green). Data have been fit to a general bimolecular equilibrium binding model to determine KD values. (D) KinExA 3000 fluorescence response to pre-equilibrated solutions of 1 nM 5-Helix-fluorescein and various concentrations of C37W628A. Solutions were mixed for 2.9 seconds prior to passage through the instrument flow cell. (E) Nonequilibrium titration of 1 nM 5-Helix-fluorescein by C37 (black), C37W628A (red) and C37L645D (green). Data have been fit to a kinetic bimolecular binding model to determine kon values. (0.38 MB PDF) [file ppat.1000674.s004.pdf]

A

5-Helix

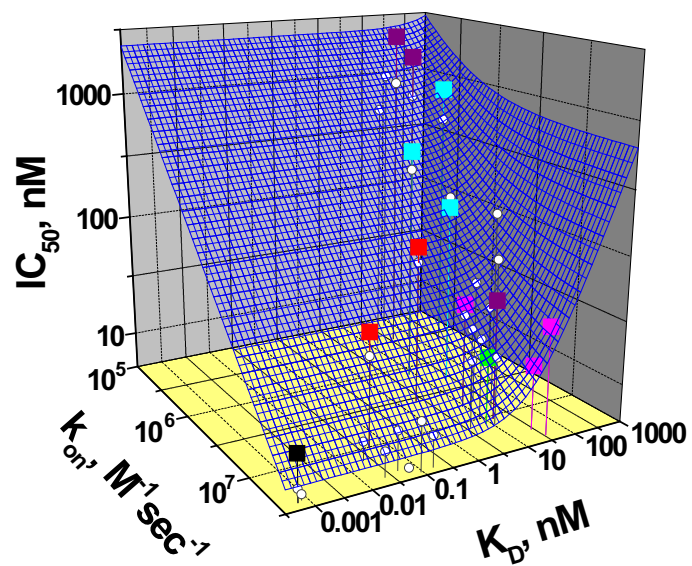

B

C37

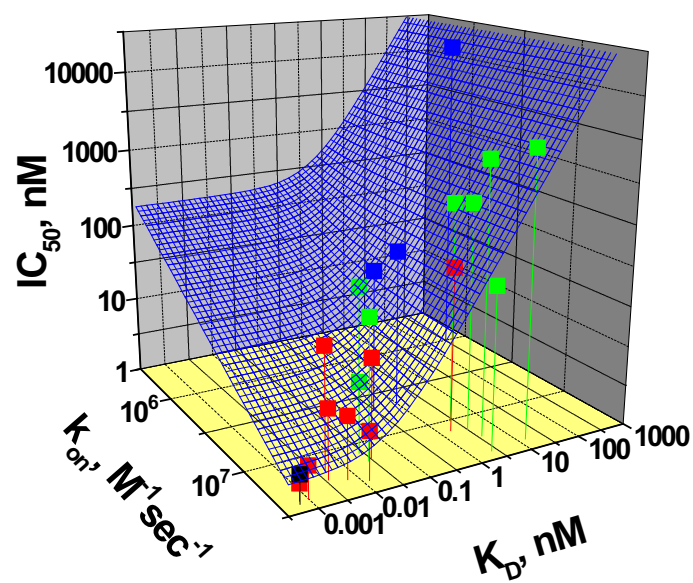

Supplement: Figure S2 — Affinity and kinetic dependence to antiviral potency. IC50 values for the 5-Helix (A) and C37 (B) variants are plotted as a function of both KD and kon. The data are color coded as in Figure 1 and globally fit to Equation 1 (blue mesh). The estimated kf and ks values are: for C37, kf = 0.054 sec−1, ks-C37 = 0.00049 sec−1; for 5-Helix, kf = 0.21 sec−1, ks-5H = 0.11 sec−1. (0.80 MB PDF) [file ppat.1000674.s005.pdf]

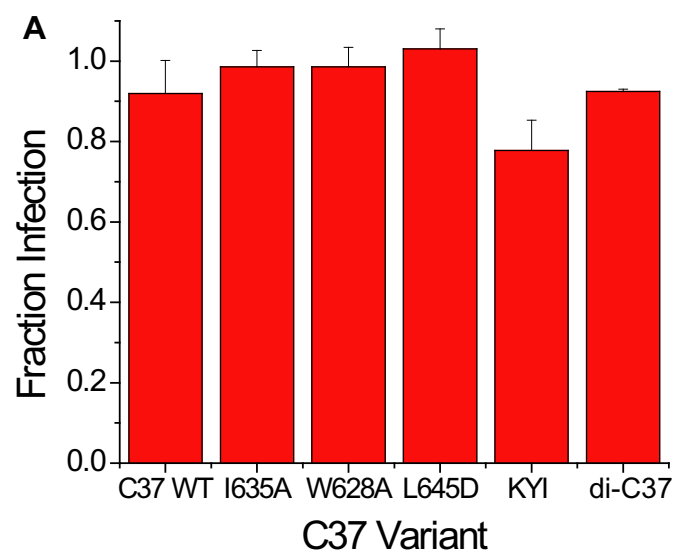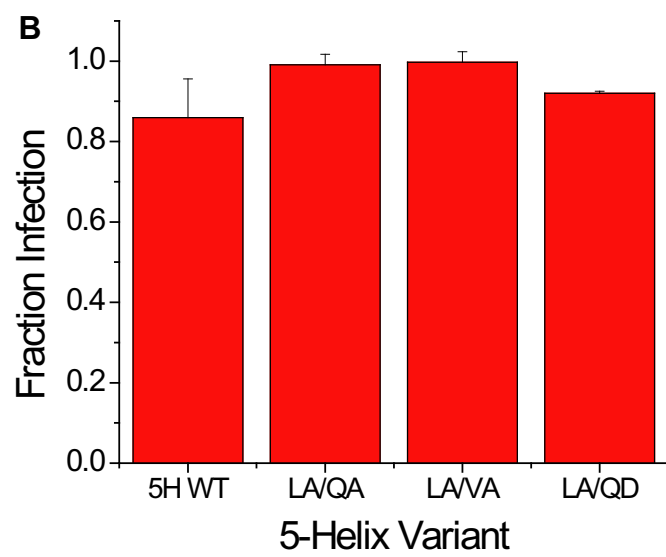

Supplement: Figure S3 — Assessment of inhibitor washout efficiency. Target cells were incubated for 2 hours with high concentrations (>IC95) of C37 variants (A) or 5-Helix variants (B) used in the inhibitor-washout viral infectivity assay. The washout procedure (3×100 µl media) was performed and cells were subsequently infected with HIV-1NL4-3 overnight. Measured viral infectivity was normalized to a no-inhibitor control. Mean values ± SEM for three independent experiments are shown. (0.20 MB PDF) [file ppat.1000674.s006.pdf]

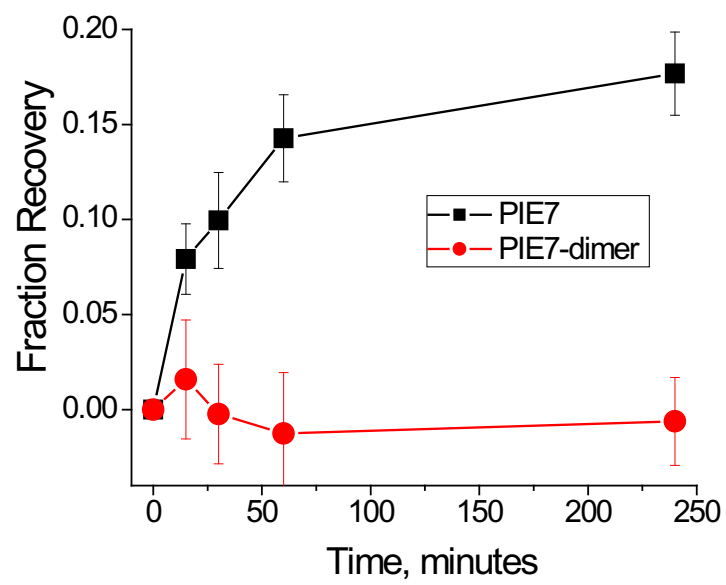

Supplement: Figure S4 — Recovery of gp41 fusion activity from PIE7 inhibition. PIE7 is a short, rigid peptide composed of D-amino acids that targets the deep hydrophobic pocket of the N-HR coiled coil [24]. A crosslinked dimer of PIE7 inhibits HIV-1 entry more potently than the monomeric form, presumably due to the enhanced binding strength afforded by multivalent interactions. The timecourse of fusion recovery from PIE7 (black) and PIE7-dimer (red) blockade was measured as described in Figure 3, except that the virus used was HIV-1HXB2 and the target cells were HOS-CD4-CXCR4. The points represent the mean ± ROM of two independent experiments. (0.18 MB PDF) [file ppat.1000674.s007.pdf]

## 5-Helix Variants

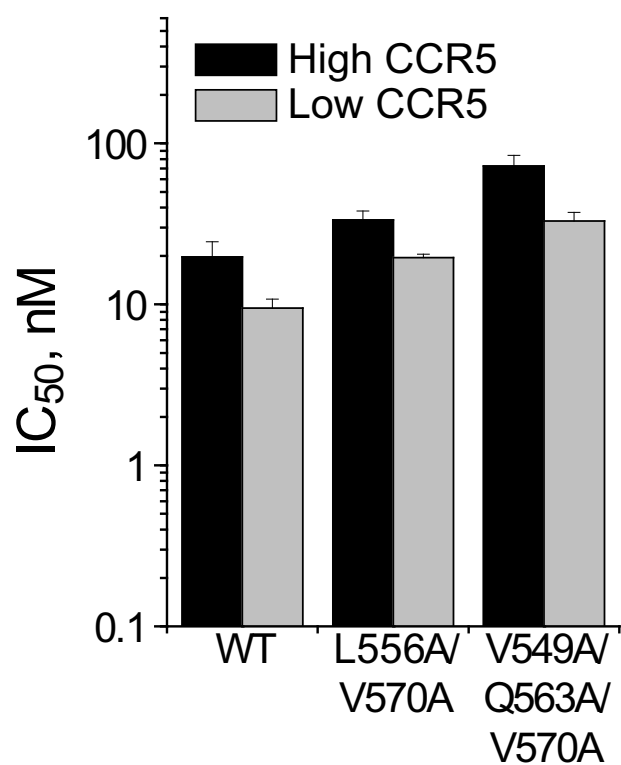

## C37 Variants

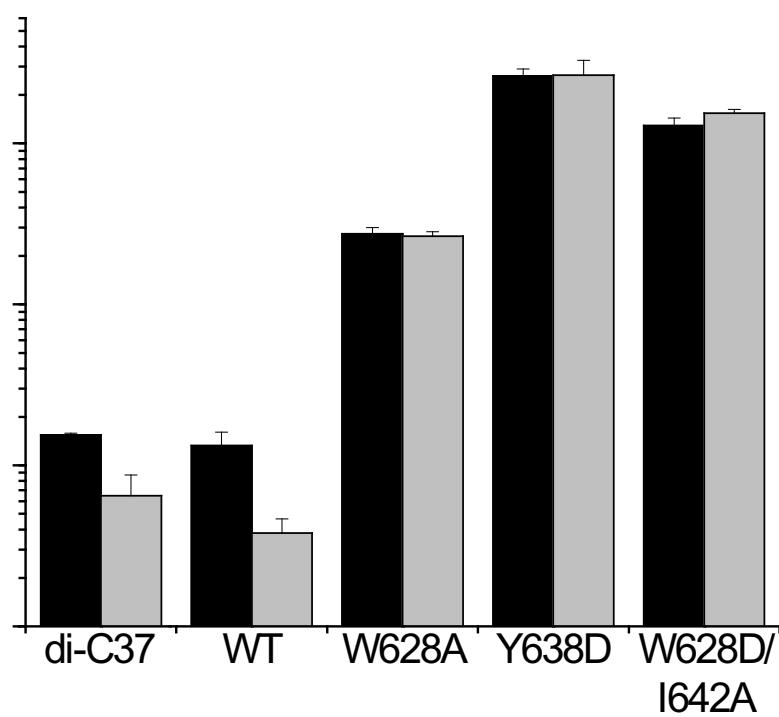

Supplement: Figure S5 — Effect of CCR5 levels on 5-Helix- and C37-inhibitory activity against HIV-1Ba-L. IC50 values were determined utilizing RC49 (black) and RC30 (gray) target cells expressing high and low levels of CCR5, respectively (see Materials and Methods). Inhibitors are ordered according to increasing koff values as measured for HXB2 sequences. The data represent the mean ± SEM of three independent experiments. (0.19 MB PDF) [file ppat.1000674.s008.pdf]

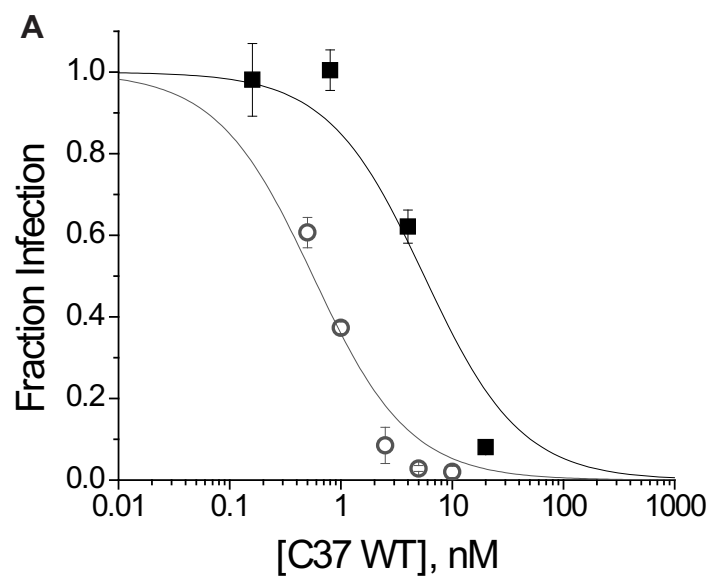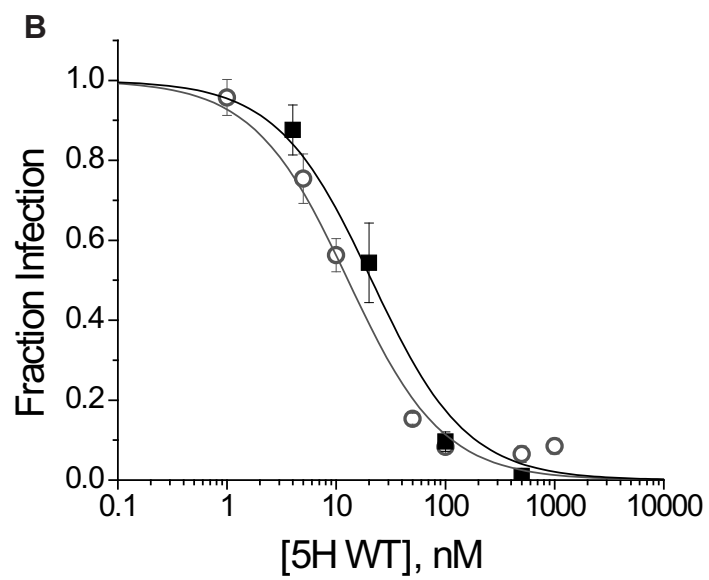

Supplement: Figure S6 — Sensitivity of inhibitor-trapped gp41 to wild type C37 and 5-Helix. (A) C37 inhibitory activity was measured by standard assay (squares) or in a 5-Helix-washout assay after Env was first trapped in the 5-HelixL556A/V570A-bound state (circles). (B) 5-Helix inhibitory activity was measured by standard assay (squares) or in a C37-washout assay after Env was first trapped in the C37W628A-bound state (circles). Experiments were conducted as described in the legend to Figure 5. (0.18 MB PDF) [file ppat.1000674.s009.pdf]

**A****Model 1**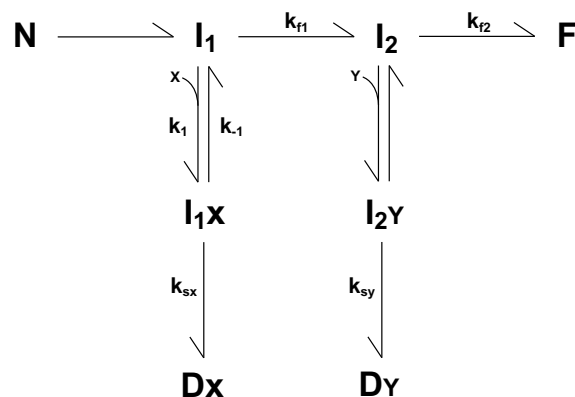**B****Model 2**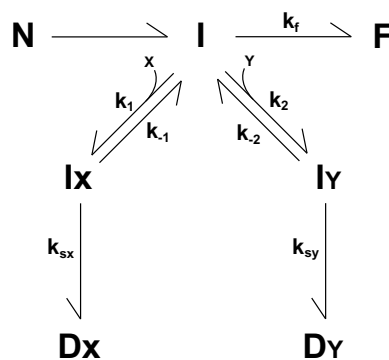**C****Model 3**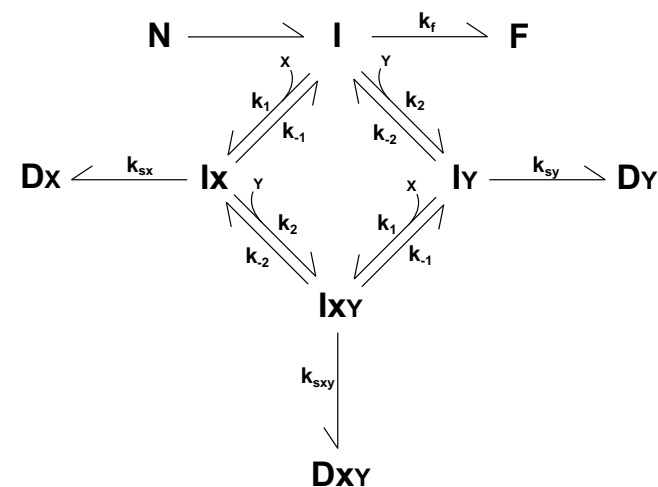**D**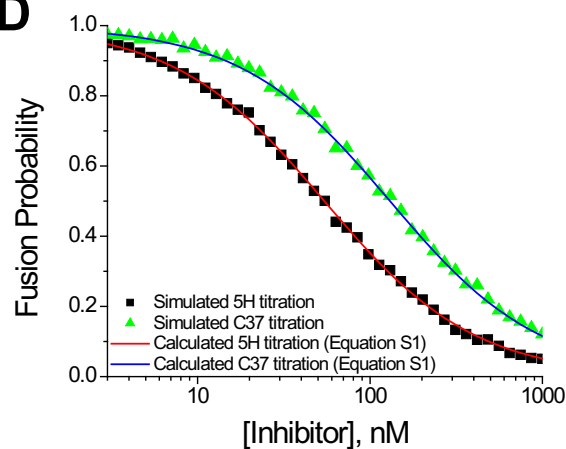**E**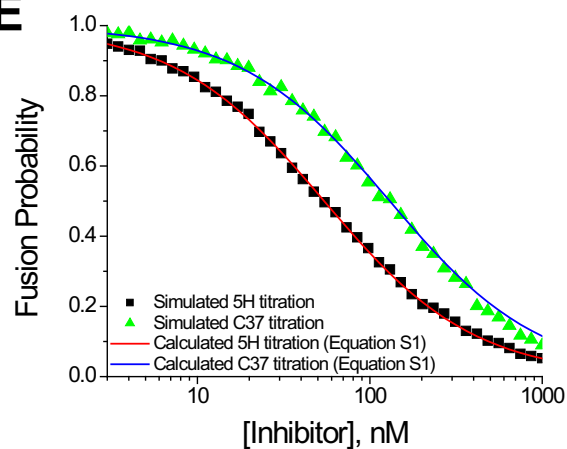**F**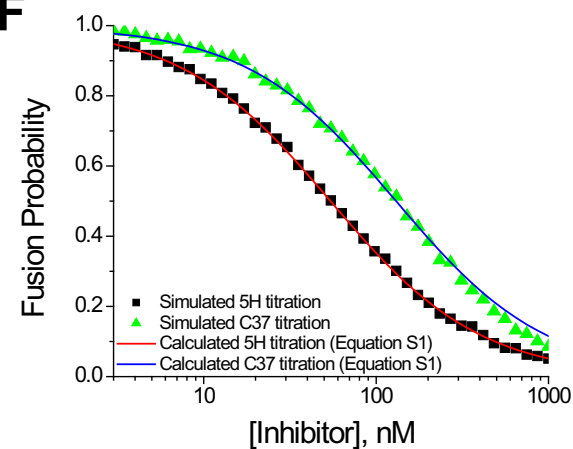**G**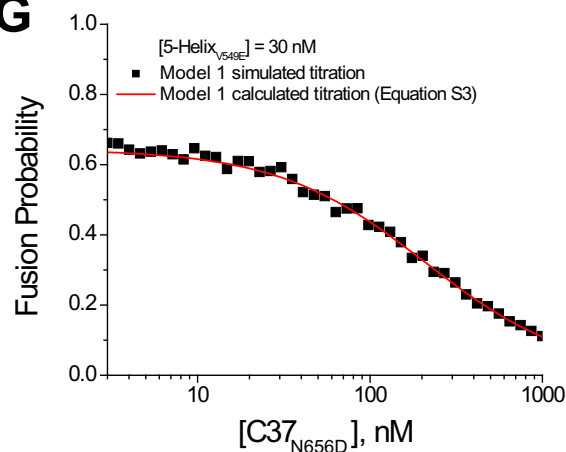**H**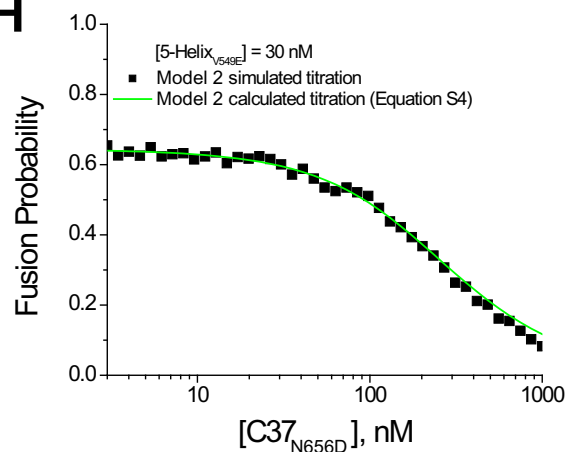**I**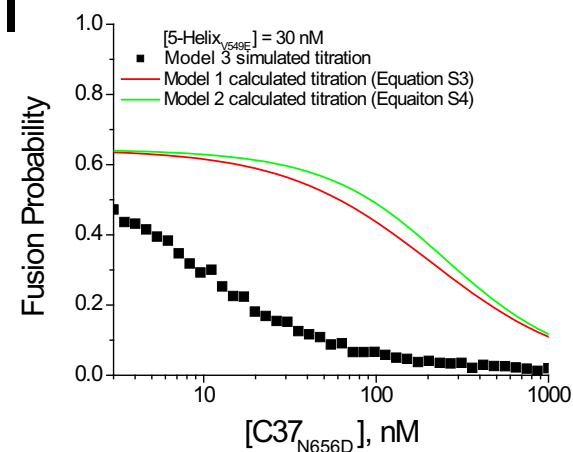

Supplement: Figure S7 — Simulating the combined inhibitory activities of 5-Helix and C37. (A, B, C) Models of intermediate state inhibition by two different inhibitors X and Y. In Model 1 (A), the inhibitors bind separate states. In Model 2 (B), the inhibitors bind separately to the same state. In Model 3 (C), the inhibitors can bind simultaneously to the same state. (D, E, F) Monte Carlo simulation of the inhibitory activities of 5-HelixV549E and C37N656D alone (symbols). The solid lines correspond to the expected titrations based upon the respective IC50 values (54 nM for 5-HelixV549E; 130 nM for C37N656D) and calculated using a Langmuir function (Equation S1, see Text S1). (G, H, I) Monte Carlo simulation of the C37N656D inhibitory activity in the presence of 30 nM 5-HelixV549E. Solid lines correspond to the analytical solution of fusion probability for Models 1 and 2 (Equations S2 and S3). Simulated points represent the average of 105 iterations. The interaction between 5-HelixV549E and C37N656D (KD = 165 nM) was taken into account for all simulations and calculations. Details of the simulation procedure and derivation of the analytical formulas are presented in Text S1. (0.25 MB PDF) [file ppat.1000674.s010.pdf]
